# Continuous, Automated Breathing Rate and Body Motion Monitoring of Rats With Paraquat-Induced Progressive Lung Injury

**DOI:** 10.3389/fphys.2020.569001

**Published:** 2020-10-16

**Authors:** Szczepan W. Baran, Ayan Das Gupta, Maria A. Lim, Ashwini Mathur, David J. Rowlands, Laura R. Schaevitz, Shiva K. Shanmukhappa, Dana B. Walker

**Affiliations:** ^1^Emerging Technologies, Laboratory Animal Services, Scientific Operations, Novartis Institutes for BioMedical Research (NIBR), Inc., Cambridge, MA, United States; ^2^Clinical Development & Analytics (CD&A), Novartis Healthcare Pvt Ltd., Hyderabad, India; ^3^Vium, Inc., San Mateo, CA, United States; ^4^Data Science and AI, Novartis Ireland Ltd., Dublin, Ireland; ^5^Respiratory Diseases, Novartis Institutes for BioMedical Research (NIBR), Inc., Cambridge, MA, United States; ^6^Preclinical Safety Assessment, Vertex Pharmaceuticals, Boston, MA, United States; ^7^Discovery Investigative Safety, Novartis Institutes for BioMedical Research (NIBR), Inc., Cambridge, MA, United States

**Keywords:** rodent, breathing rate, activity, paraquat, lung injury, drug discovery, translational research, digital biomarkers

## Abstract

Assessments of respiratory response and animal activity are useful endpoints in drug pharmacology and safety research. We investigated whether continuous, direct monitoring of breathing rate and body motion in animals in the home cage using the Vum Digital Smart House can complement standard measurements in enabling more granular detection of the onset and severity of physiologic events related to lung injury in a well-established rodent model of paraquat (PQ) toxicity. In rats administered PQ, breathing rate was significantly elevated while body motion was significantly reduced following dosing and extending throughout the 14-day study duration for breathing rate and at least 5 days for both nighttime and daytime body motion. Time course differences in these endpoints in response to the potential ameliorative test article bardoxolone were also readily detected. More complete than standard in-life measurements, breathing rate and body motion tracked injury progression continuously over the full study time period and aligned with, and informed on interval changes in clinical pathology. In addition, breathing rates correlated with terminal pathology measurements, such as normalized lung weights and histologic alveolar damage and edema. This study is a preliminary evaluation of the technology; our results demonstrate that continuously measured breathing rate and body motion served as physiologically relevant readouts to assess lung injury progression and drug response in a respiratory injury animal model.

## Introduction

The ability to measure pulmonary response in preclinical studies is important not only in respiratory disease models but also in safety pharmacology and toxicology studies (Murphy, 2002). Common methods of assessing such in laboratory animals include direct pulmonary function testing such as spirometry or indirect measurements based on clinical pathology parameters and pulmonary histopathology. All these methods are discontinuous measures, highly limited in the frequency of study application, and with a significant degree of invasiveness and personnel resources. To overcome these time course and handling/interventional limitations, we evaluated a home cage monitoring system that incorporates continuous, automated readouts of breathing rate and whole-body motion (as a measure of activity) in individual animals.

To assess the utility of this home cage system, we applied it to a rat model of paraquat (PQ)-induced lung injury (PQiLI). This model is a well-described and commonly used rodent model of pulmonary fibrosis bearing some histopathological similarity to Idiopathic Pulmonary Fibrosis (IPF) due to its rapid injury onset (Silva and Saldiva, 1998; Tomita et al., 2007; Sun and Chen, 2016). IPF is a progressive fatal interstitial lung disease characterized by fibrosis of the lungs resulting in shortness of breath and reduced exercise capacity (Ley et al., 2011). Animal models that replicate features of IPF play an important role in understanding the pathogenic mechanisms of relevant respiratory diseases and in developing novel therapies (Matute-Bello et al., 2008). PQ was originally developed as an herbicide and selectively accumulates in the lungs by inhalation, ingestion, or physical contact, resulting in severe lung injury in humans. Several of the physiological and pathological features in the development of IPF are present in rodents exposed to PQ. These features include time course changes from lung edema and interstitial inflammation to progressive fibrosis (Silva and Saldiva, 1998; Tomita et al., 2007; Sun and Chen, 2016). The PQiLI model has been used to evaluate safety and efficacy of therapies used to treat lung injury and fibrosis (Santos et al., 2011; Chen et al., 2013a,b, 2015; Liu et al., 2013; Qian et al., 2014; Shao et al., 2015).

Currently, animal models of IPF, including PQiLI, are evaluated using indirect measures of clinical observations, body weight, body temperature, and clinical laboratory measurements. In addition, histopathological changes of terminal lung specimens are commonly assessed (Costa et al., 2014; Shao et al., 2015; Rumsey et al., 2017). Pulmonary function endpoints such as plethysmography or forced oscillation are not a routine part of preclinical assessments or commonly utilized, likely due to the technical challenges of executing these types of methods (Jenkins et al., 2017). The fairly invasive forced oscillation technique requires anesthesia and is often terminal (Bates and Irvin, 2003; Vanoirbeek et al., 2010; Hoymann, 2012). On the other hand, the less invasive unrestrained plethysmography involves placing awake, freely moving animals into a restrictive chamber for monitoring. This requires additional time for habituation in order to reduce inter-and intra-subject variability as well as possible stress-related effects on pulmonary function measurements (Hoymann, 2012; Lim et al., 2014; Quindry et al., 2016). All of these methods, both the common tests and advanced functional assessments, are constrained by the frequency of data collection, impacted by artifacts of animal handling, and for some, limited by its invasiveness or need for animal euthanasia. These methods thus provide only interrupted measurement of the progression and/or severity of the injury during the course or end of study. These limitations underscore the need for more longitudinal monitoring of animals in their natural state using novel, home cage systems to continuously assess measures of movement, specifically breathing rate, and body motion in rodent models. Measuring body motion may be akin to measuring exercise capacity by the clinical 6-min walk test as a prognostic indicator in IPF patients, recognizing that there are some differences as the rats may not reach maximal exertion (Ley et al., 2011; Du Bois et al., 2014).

Here, we evaluate whether monitoring of animal breathing rate and body motion in the home cage can serve as useful digital readouts, complementary to routine measures for assessing injury progression in PQiLI. These digital readouts were compared to standard endpoint measurements (e.g., body weight, body temperature, clinical pathology, and histopathology) collected at three different post-dose time points, representing acute, subacute, and chronic stages. We used a potentially ameliorative therapy administration of bardoxolone, as a tool to assess sensitivity of these digital readouts in a standard preclinical pharmacological study.

## Materials and Methods

### Animals

Thirteen week-old, male Lewis rats, (Charles River Laboratories, Hollister, CA, United States), were used in these studies. Upon arrival, rats were single-housed in instrumented individually ventilated cages (IVC; 17.0 × 13.4 × 7.8 in; Digital Smart House, Vium, San Mateo, CA, United States and Innovive, San Diego, CA, United States) containing corncob bedding, *ad libitum* access to food (Pico Rodent Diet 20 EXT IRR, LabDiet, Inc., St. Louis, MO, United States), and sterile water (Innovive, San Diego, CA, United States). Animals were maintained in a specific-pathogen-free (SPF) facility under a 12-h light-dark cycle (06:00–18:00 PDT) with uniform, cage-level light emitting diode (LED) illumination, temperature, and humidity. Experiments were conducted in Vium’s American Association for Laboratory Animal Care (AAALAC)-accredited Digital Vivarium in accordance with the current National Research Council *Guide for the Care and Use of Laboratory Animals* and were approved by Vium’s Institutional Animal Care and Use Committee.

### Test Articles

Test articles are listed in Table 1. PQ (Sigma, St. Louis, MO, United States) was dissolved in sterile saline^1^ (Molecular Biologicals International, Inc., Irvine, CA, United States). Sterile saline was used as control material for the intra-tracheal instillation procedure. Bardoxolone (Sigma, St. Louis, MO, United States or Cayman Chemical Company, Ann Arbor, MI, United States) was dissolved in sterile water containing 1% Methylcellulose (MC; 400 cp; Sigma, St. Louis, MO, United States). Sterile water containing 1% MC was used as control material for oral dosing.

**Table 1:** Study groups table.

Study group#	Study end (days)	Actual sample size^a^	Injury induction agent	Therapeutic agent	Treatment regimen	Study group name
1	3	8	Saline	Vehicle 1% Methylcellulose (400 cp)	Prophylactic	SA/Veh/P
	6	8				
	14	6^b^				
2	3	8	Paraquat (0.06 mg/kg)	Vehicle 1% Methylcellulose (400 cp)	Prophylactic	PQ/Veh/P
	6	8				
	14	7^c^				
3	3	8	Paraquat (0.06 mg/kg)	Bardoxolone (10 mg/kg/PO)	Prophylactic	PQ/Bar/P
	6	8				
	14	8				
4	3	8	Paraquat (0.06 mg/kg)	Vehicle 1% Methylcellulose (400 cp)	Therapeutic	PQ/Veh/T
	6	8				
	14	8				
5	3	8	Paraquat (0.06 mg/kg)	Bardoxolone (10 mg/kg/PO)	Therapeutic	PQ/Bar/T
	6	8				
	14	7^d^				

a The intended sample size for each group was 8.

b One rat died shortly after induction, and another rat was excluded from all subsequent analysis due to the presence of symptoms that were suspect to be resulting from inadvertent PQ administration.

c One rat died shortly after induction.

d One rat was euthanized prior to scheduled endpoint.

### Paraquat-Induced Lung Injury Model

Animals were allowed to acclimatize for 14 days in Vium Digital Smart Houses. Animals were assigned into the following study groups: control rats administered saline and vehicle (SA/Veh/P), rats administered PQ and vehicle prophylactically or therapeutically (PQ/Veh/P and PQ/Veh/T, respectively or PQ/Veh collectively), and rats administered PQ and bardoxolone prophylactically and therapeutically (PQ/Bar/P and PQ/Bar/T, respectively or PQ/Bar collectively; see Table 1 for detailed information on study groups). A stratified randomization method was used to ensure that all study groups had similar average baseline body weights and activity profiles prior to beginning of study.

**Figure 1:**
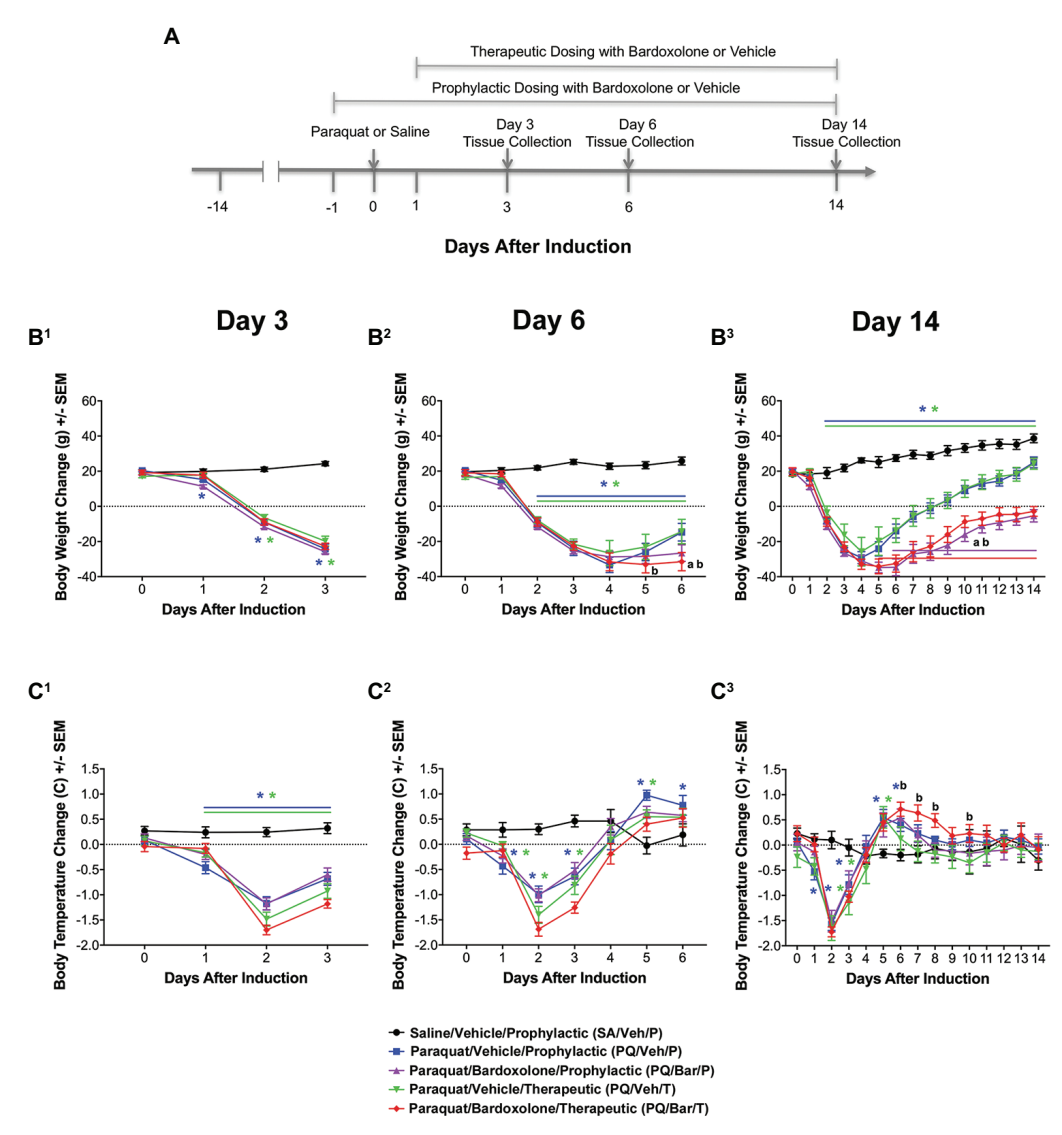
Standard in-life measurements confirmed lung injury and detected effects of bardoxolone treatment in a rodent model of paraquat (PQ)-induced lung injury (PQiLI). **(A)** Experimental timeline. After a 14-day acclimation period, rats were given an intra-tracheal dose of PQ on Day 0. Rats were orally treated with bardoxolone or vehicle either prophylactically starting the day before PQ administration (Day 1) or therapeutically starting the day after PQ administration (Day 1) daily until study end. Treatment schedules are shown in brackets. Three separate subgroups of rats per study group were euthanized on either Days 3, 6, or 14 for endpoint tissue collection. Automated breathing rate and body motion readouts were collected continuously throughout the study, and standard in-life measurements (body weight and body temperature) were collected daily during the study. **(B)** Change in body weight. Compared with control rats (SA/Veh/P), body weight was decreased in PQ/Veh/P and PQ/Veh/T rats. These changes were observed as early as Day 1 and as late as Day 14. Bardoxolone-treated rats (PQ/Bar/P and PQ/Bar/T) showed lower body weights compared with vehicle-treated counterparts. ^*^p ≤ 0.05 vs. SA/Veh/P. ^a^*p* ≤ 0.01 PQ/Veh/P vs. PQ/Bar/P, and ^b^*p* ≤ 0.01 PQ/Veh/T vs. PQ/Bar/T. **(C)** Change in body temperature. Compared with SA/Veh/P rats, body temperature was decreased in PQ/Veh/P and PQ/Veh/T rats as early as Day 1 and as late as Day 3. Despite showing an earlier decrease in body temperature, PQ/Bar/T rats demonstrated higher body temperatures on Days 6–8 and Day 10. **(B^1^,C^1^)** Day 3 (*n* = 24 rats for all study groups except *n* = 23 for SA/Veh/P). **(B^2^,C^2^)** Day 6 (*n* = 16 rats for all study groups). **(B^3^,C^3^)** Day 14 (*n* = 8 rats for all study groups except *n* = 7 for SA/Veh/P). ^*^p ≤ 0.05 vs. SA/Veh/P. ^a^*p* ≤ 0.05 PQ/Veh/P vs. PQ/Bar/P and ^b^*p* ≤ 0.05 PQ/Veh/T vs. PQ/Bar/T. Error bars are +/− SEM.

Rats were anesthetized under isoflurane, and non-control animals were administered a single 0.3 ml intra-tracheal dose of PQ (0.06 mg/kg; Silva and Saldiva, 1998; Sun and Chen, 2016). Control rats were administered a single intra-tracheal dose of the same volume of saline. For all individual animals, body weight was measured daily, and body temperature was measured continuously using radio-frequency identification (RFID) microchips (BioMedic Data Systems, Inc., Seaford, DE, United States) implanted 7 days prior to intra-trachael PQ-instillation. Animals were immediately euthanized if criteria for humane endpoint were met. Figure 1A illustrates a schematic of the study design.

### Treatment With Bardoxolone

Two of the four groups of rats administered PQ were treated orally (PO) with bardoxolone (10 mg/kg; Groups 3 and 5 in study groups table), while the rest of the groups were treated orally with sterile saline containing 1% MC as control (vehicle; Groups 1, 2, and 4 in study groups table). Rats given bardoxolone were divided into two treatment groups: prophylactic or therapeutic. For the prophylactic treatment group, bardoxolone was administered once 24 h prior to study (Day 1), then an hour prior to PQ instillation, followed by daily administration until the end of the study period. For the therapeutic treatment group, bardoxolone was administered 24 h post-PQ instillation and continued daily until the end of the study period. All experimenters were blinded to treatment groups during the study for collection of in-life data.

### The Vium Digital Platform

As described elsewhere (Lim et al., 2017, 2019; Do et al. accepted in Comparative Medicine), Vium Digital Smart Houses consist of standard IVC slotted in Vium’s rack system. The Digital Smart Houses are outfitted with sensors and high-definition (HD) cameras that enable continuous, 24/7 monitoring of animals that streams data to a secure cloud-based infrastructure. Video is processed using computer vision algorithms to produce a digital history of body motion (mm/s) and breathing rate (breaths per min or bpm; Lim et al., 2017, 2019; Do et al. accepted in Comparative Medicine). Briefly, to compute body motion, computer vision algorithms use visible and infrared spectrum video captured by an HD camera at 24 frames per second. Standard optical flow algorithms (Luo et al., 2016) were used to quantify the motion of the animal in the cage with movement read out at 24 frames/s as speed (mm/s). To compute breathing rate, computer vision algorithms search for >30 s regions of time when animals are stationary and identify periodic body motion in the animal’s chest/thoracic area that falls within a frequency band containing known rodent breathing rates (Groeben et al., 2003). The peak root mean square (RMS) power across a 30 s time window is compared to a threshold to determine whether the periodic body motion is significant. When periodic motion is significant, the frequency is read out as the breathing rate. On average, the algorithms capture ~400 breathing rate points per day with 75% of rates captured during the day and 25% captured at night when the animals are more active. The ability of computer vision to accurately monitor breathing rate by comparing breathing rates generated by the computer to those captured from awake freely moving animals in a whole body plethysmograph (see the section Supplementary Materials and Supplementary Figure 1).

### Clinical Pathology and Histopathology

Anatomic and clinical pathology data were evaluated by appropriate veterinary board certified pathologists. A subset of animals from each study group (*n* = 6–8/group) were euthanized on Days 3, 6, and 14 (Table 1; Figure 1A). Animals were euthanized by isoflurane inhalation followed by exsanguination by cardiac puncture for terminal blood collection. Blood samples for hematology were collected in ethylenediaminetetraactetic acid (EDTA) tubes and stored at 2–8°C for no more than 4 h. Blood samples for clinical chemistry were collected without anticoagulant and processed to serum that was stored at ≤−70°C until analysis. Hematology was performed using the Abaxis VetScan HM5 (Abaxis, Inc., Union City, CA, United States), and serum chemistry analysis was performed using the Cobas® c501 Analyzer (Roche Diagnostics, Basel, Switzerland). Lungs were fixed in 10% buffered neutral formalin (pH 7.4), processed routinely for embedding in paraffin, sectioned at 4 μm, and then stained with Hematoxylin and Eosin (H&E). Histologic severity grades were assigned to findings during a subjective evaluation of the H&E-stained lung sections using light microscopy. The following severity grades were assigned: minimal = 1, mild = 2, moderate = 3, and severe = 4.

### Data and Statistical Analysis

#### Continuous Physiologic Data Normalization

Raw body daytime motion [collected from 06.00 to 18.00 Pacific Daylight Time (PDT)], nighttime motion (collected from 18.00 to 06.00 PDT), and breathing rate metrics (collected from 06.00 to 06.00 PDT) were separately averaged daily for each study day. To calculate the change in body motion and breathing rate for each study day (main study phase), raw metrics from Day 5 to Day 2 prior to PQ instillation (acclimation phase) were first averaged to create an average baseline value (see Supplementary Figure 1). Next, this averaged baseline value was subtracted from the average daytime body motion, nighttime body motion, or breathing rate collected for each study day. To calculate the change in body weight and body temperature for each study day, measurements gathered on Day 7 prior to PQ instillation were subtracted from the body weight and body temperature measurements gathered during each study day.

#### Sample Sizes and Subject Exclusion

The sample size of eight animals per study group for each time point (Table 1) was determined based on data from previously published literature (Zhang et al., 2013). Based on the observed baseline breathing rate of 107 bpm for the control group (SA/Veh/P) and a pooled standard deviation of 13, we determined that this study will have more than 95% power to detect a 30% increase from baseline for breathing rate results. For all Day 3 body weight, body temperature, body motion, and breathing rate data, results were combined for all animals within each treatment group (*n* = 23–24 rats/group). For all Day 6 data, results were combined for all animals surviving to this time point within each treatment group (*n* = 16 rats/group). For all Day 14 data, results were used only for animals surviving to this time point (*n* = 7–8 rats/group). For clinical pathology, lung weights, and histopathology analyses, data for each time point were analyzed separately (*n* = 6–8 rats/group). One animal from the SA/Veh/P study group was excluded from all subsequent analysis due to the presence of symptoms that were suspect to be resulting from inadvertent PQ administration.

#### Statistical Tests

For body weight, body temperature, breathing rate, and body motion data, mixed model repeated measures (MMRM) ANOVA was fitted for Day 3 and Day 6 analyses, while an ANCOVA was fitted for Day 14 analyses to compare the effects of treatment group and time (study day). Follow-up pairwise comparisons were made using *t*-tests for comparing between groups.

For clinical pathology, two-way ANOVAs followed by Dunnett’s or Cochran and Cox tests, as applicable, were used to compare PQ/Veh/P and PQ/Bar/P with SA/Veh/P control rats. Similar tests were used to compare PQ/Bar/T with PQ/Veh/T. For lung weight normalized to endpoint body weight, a one-way ANOVA followed by Tukey’s test was used to compare between treatment groups. For histopathology scoring, one-way ANOVAs followed by Dunnett’s tests were used to compare both PQ/Bar/P and PQ/Bar T rats to PQ/Veh/P rats since PQ/Veh/T tissue samples did not undergo histopathology scoring. Although SA/Veh/P tissue samples underwent histopathology scoring, this data were not analyzed because all scores were “0.” Pearson’s correlation tests and linear regression analyses were used to investigate the relationship between final day breathing rate and normalized lung weights or histopathology scores. Values of *p* < 0.05 were considered statistically different. SAS 9.4 (SAS Software, Cary, NC, United States) was used for body weight, body temperature, body motion, and breathing rate statistical analyses. Pristima 7.4 (Xybion, Lawrenceville, New Jersey) was used for clinical chemistry and hematology analyses, while Prism 8.0 (GraphPad Software, La Jolla, CA) was used for all other statistical analyses.

## Results

We investigated the utility of a continuous monitoring platform and its automated breathing rate and body motion readouts for assessing acute and chronic toxic injury progression in rats post-intra-tracheal PQ administration and compared these digital readouts with standard endpoint measurements. All animals survived to their scheduled euthanasia time point with the exception of one rat from each of the following groups: SA/Veh/P, PQ/Veh/P, and PQ/Bar/T. The first two rats died shortly post-intra-tracheal dosing on induction day (Day 0), and the third was humanely euthanized on Day 8 after reaching ≥20% body weight loss.

### Body Weight Decreased Post-PQ Administration, and Showed Relative Delayed Return to Baseline With Bardoxolone Treatment

Compared with SA/Veh/P rats, rats administered PQ (PQ/Veh/P, PQ/Veh/T, PQ/Bar/P, and PQ/Bar/T) showed lower body weights as early as Day 1 and as late as Day 14 (*p* ≤ 0.05 for both PQ/Veh groups and *p* ≤ 0.001 for both PQ/Bar groups; Figure 1B). PQ/Bar/P rats weighed less than PQ/Veh/P rats on Day 6–14, and PQ/Bar/T rats weighed less than PQ/Veh/T rats on Days 5–14 (*p* ≤ 0.01 for both comparisons; Figures 1B^2^,B^3^).

### Body Temperature Transiently Decreased Post-PQ Administration With No Effect of Bardoxolone Treatment

Compared with SA/Veh/P rats, rats administered PQ (PQ/Veh/P, PQ/Veh/T, PQ/Bar/P, and PQ/Bar/T) had lower body temperatures as early as Day 0 and as late as Day 3 and higher body temperatures as early as Day 5 and as late as Day 8 (*p* ≤ 0.05 for all groups; Figure 1C). PQ/Bar/P rats had similar body temperatures to PQ/Veh/P rats throughout the study. In contrast, PQ/Bar/T rats had higher body temperatures compared with PQ/Veh/T rats on Days 6–8, and Day 10 (*p* ≤ 0.05; Figure 1C^3^).

### Breathing Rate Increased Post-PQ Administration and Showed Attenuation With Prophylactic, but Not Therapeutic Bardoxolone Treatment

**Figure 2:**
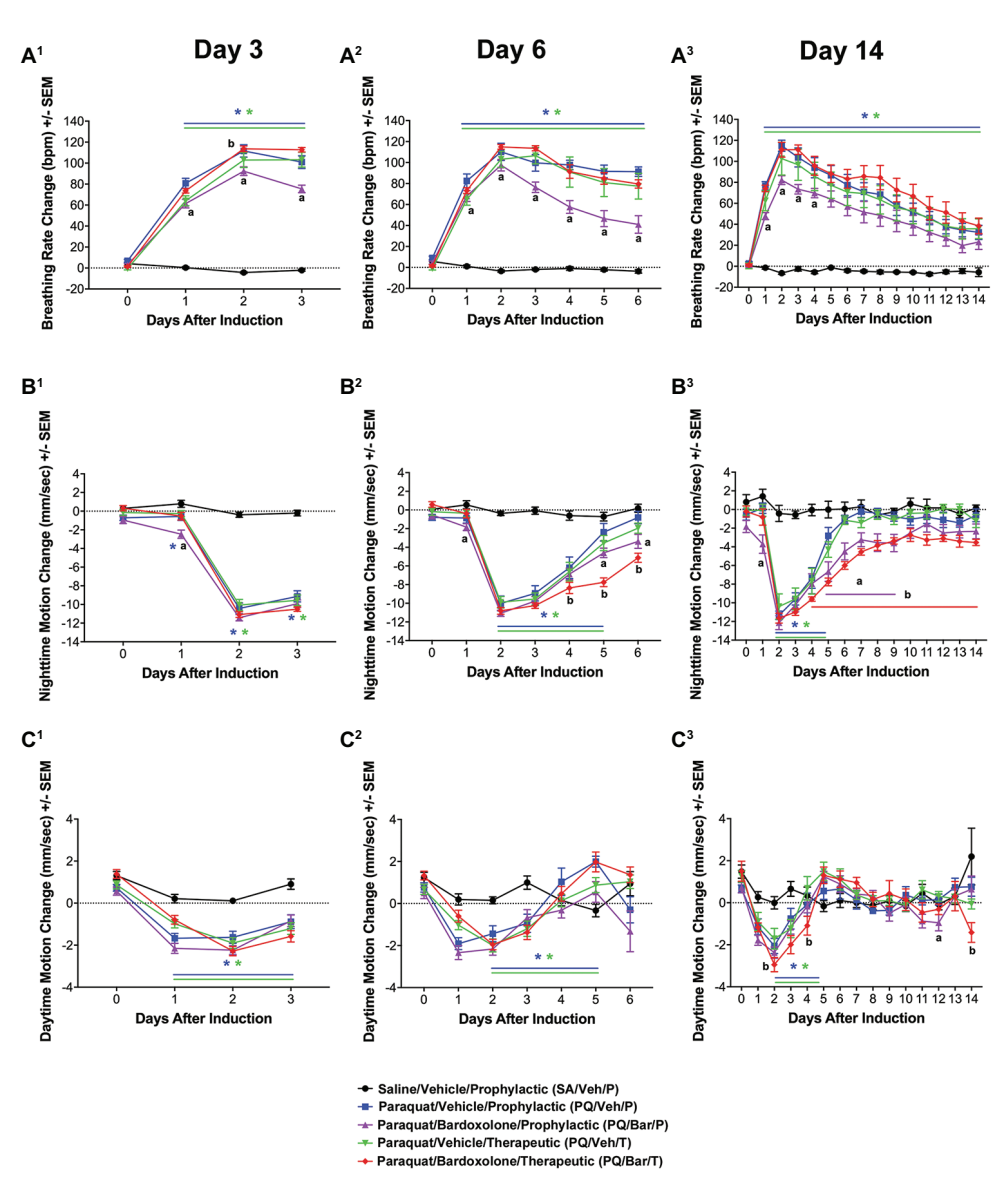
Automated breathing rate and body motion detected injury and effects of bardoxolone in a rodent model of PQiLI. **(A)** Change in breathing rate. Compared with control rats (SA/Veh/P), breathing rate was increased in PQ/Veh/P and PQ/Veh/T rats. Elevated breathing rate was observed as early as Day 1 and as late as Day 14. PQ/Bar/P, but not PQ/Bar/T, rats had lower breathing rates compared with vehicle-treated counterparts. ^*^p ≤ 0.01 vs. SA/Veh/P, ^a^*p* ≤ 0.05 PQ/Veh/P vs. PQ/Bar/P, and ^b^*p* ≤ 0.05 PQ/Veh/T vs. PQ/Bar/T. **(B)** Change in nighttime body motion. Compared with SA/Veh/P rats, nighttime body motion was decreased in PQ/Veh/P and PQ/Veh/T rats as early as Day 1 and as late as Day 5. The recovery of nighttime body motion in PQ/Bar/P and PQ/Bar/T rats was delayed. ^*^p ≤ 0.01 vs. SA/Veh/P. ^a^*p* ≤ 0.05 PQ/Veh/P vs. PQ/Bar/P and ^b^*p* ≤ 0.01 PQ/Veh/T vs. PQ/Bar/T. **(C)** Change in daytime body motion. Compared with SA/Veh/P rats, daytime body motion was decreased in PQ/Veh/P and PQ/Veh/T rats as early as Day 1 and as late as Day 12. PQ/Bar/P and PQ/Bar/T rats showed lower daytime body motion compared with vehicle-treated counterparts during specific days. **(A^1^,B^1^,C^1^)** Day 3 (*n* = 24 rats for all study groups except *n* = 23 for SA/Veh/P). **(A^2^,B^2^,C^2^)** Day 6 (*n* = 16 rats for all study groups). **(A^3^,B^3^,C^3^)** Day 14 (*n* = 8 rats for all study groups except *n* = 7 for SA/Veh/P). ^*^p ≤ 0.05 vs. SA/Veh/P. ^a^*p* ≤ 0.05 PQ/Veh/P vs. PQ/Bar/P and ^b^*p* ≤ 0.05 PQ/Veh/T vs. PQ/Bar/T. Error bars are +/− SEM.

Pre-induction, average breathing rate levels were similar among study groups (Supplementary Figure 2). Beginning 16 h after induction, breathing rates were consistently increased for all groups administered PQ compared with SA/VEH/P (*p* ≤ 0.0001; Supplementary Figure 3). Given the similar injury response to PQ across groups, further analysis focused on daily averages. Post-induction, PQ/Veh/P, PQ/Veh/T, PQ/Bar/P, and PQ/Bar/T rats demonstrated higher breathing rates on Days 1–14 (*p* ≤ 0.01 for both PQ/Veh groups and *p* ≤ 0.05 for both PQ/Bar groups compared with SA/Veh/P; Figure 2A). PQ/Bar/P rats demonstrated lower breathing rates compared with PQ/Veh/P rats as early as Day 1 and as late as Day 6 (*p* ≤ 0.05; Figure 2A). In contrast, PQ/Bar/T rats demonstrated even higher breathing rates at Day 2 compared with PQ/Veh/T rats (*p* ≤ 0.05; Figure 2A).

### Nighttime Body Motion Transiently Decreased Post-PQ Administration, and Bardoxolone Treatment Delayed Return to Baseline

Pre-induction, average nighttime body motion levels were equivalent among groups (Supplementary Figure 1). Post-induction, PQ/Veh/P, PQ/Veh/T, PQ/Bar/P, and PQ/Bar/T rats showed lower nighttime body motion between Day 2 and Day 5 (*p* ≤ 0.01 for both PQ/Veh groups and *p* ≤ 0.05 for both PQ/Bar groups compared with SA/Veh/P; Figure 2B). PQ/Bar/P and PQ/Bar/T rats also displayed a delayed return to baseline body motion (*p* ≤ 0.05 for both groups compared with SA/Veh/P). PQ/Bar/P rats were less active than PQ/Veh/P rats on Day 1 and Days 5–9, while PQ/Bar/T rats were less active than PQ/Veh/T rats on Days 4–14 (*p* ≤ 0.05 and *p* ≤ 0.01, respectively; Figure 2B).

### Daytime Body Motion Transiently Decreased Post-PQ Administration With No Effect of Bardoxolone Treatment

Pre-induction, average daytime body motion levels were similar among groups (Supplementary Figure 1). Post-induction, PQ/Veh/P and PQ/Veh/T rats showed lower daytime body motion as early as Day 1 and as late as Day 5 (*p* ≤ 0.05 for both groups compared with SA/Veh/P; Figure 2C). PQ/Bar/P and PQ/Bar/T rats displayed lower daytime body motion with generally earlier onset and over a longer time course: as early as Day 0 and as late as Day 14 (*p* ≤ 0.01 for both groups compared with SA/Veh/P). PQ/Bar/P rats were less active than PQ/Veh/P rats on Day 12, and PQ/Bar/T rats were less active than PQ/Veh/T rats on Days 2, 4, and 14 (*p* ≤ 0.05 for both comparisons; Figure 2C).

### Clinical Pathology Measurements Showed Changes Post-PQ Administration and Response to Bardoxolone Treatment

**Figure 3:**
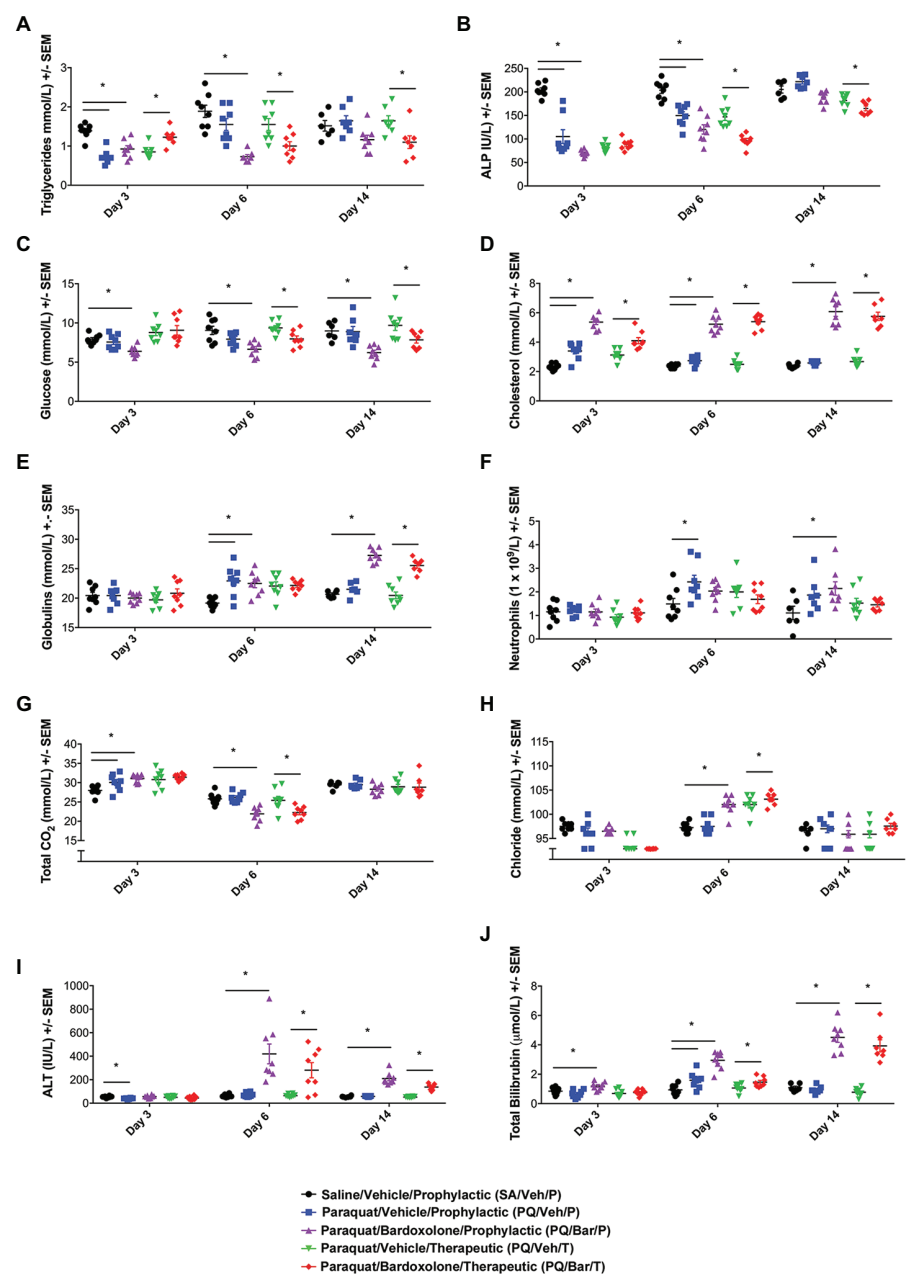
Clinical pathology performed at distinct time points showed changes in response to PQ administration and bardoxolone treatment. **(A)** Triglycerides. PQ/Veh/P rats showed lower triglyceride levels on Day 3 compared with SA/Veh/P. Bardoxolone-treated rats (PQ/Bar/P and PQ/Bar/T) demonstrated changes as early as Day 3 and as late as Day 14. **(B)** Alkaline Phosphatase (ALP). PQ/Veh/P rats showed lower ALP levels on Days 3 and 6 compared with SA/Veh/P. PQ/Bar/P and PQ/Bar/T rats demonstrated lower levels as early as Day 3 and as late as Day 14. **(C)** Glucose. SA/Veh/P and PQ/Veh/P rats showed similar glucose levels at all evaluated time points. In contrast, PQ/Bar/P and PQ/Bar/T rats demonstrated lower levels as early as Day 3 and as late as Day 14. **(D)** Cholesterol. PQ/Veh/P rats showed higher cholesterol levels on Days 3 and 6 compared with SA/Veh/P. PQ/Bar/P and PQ/Bar/T rats demonstrated higher levels at all evaluated time points. **(E)** Globulins. PQ/Veh/P rats showed higher globulin levels on Day 6 compared with SA/Veh/P. PQ/Bar/P and PQ/Bar/T rats demonstrated higher levels as early as Day 6 and as late as Day 14. **(F)** Neutrophils. PQ/Veh/P rats showed higher neutrophil levels on Day 6 compared with SA/Veh/P. PQ/Bar/P rats demonstrated higher levels on Day 14. **(G)** Total CO_2_ or bicarbonate. PQ/Veh/P rats showed higher total CO_2_ levels on Day 3 compared with SA/Veh/P. PQ/Bar/P rats demonstrated higher levels on Day 3 and lower levels on Day 6. PQ/Bar/T rats demonstrated lower levels on Day 6. **(H)** Chloride. SA/Veh/P and PQ/Veh/P rats showed similar chloride levels at all evaluated time points. PQ/Bar/P and PQ/Bar/T rats demonstrated higher levels on Day 6. **(I)** Alanine transaminase (ALT). PQ/Veh/P rats showed lower ALT levels on Day 3 compared with SA/Veh/P. PQ/Bar/P and PQ/Bar/T rats demonstrated higher levels on Days 6 and 14. **(J)** Total Bilirubin. PQ/Veh/P rats showed higher total bilirubin levels on Day 6 compared with SA/Veh/P. PQ/Bar/P and PQ/Bar/T rats demonstrated higher levels as early as Day 3 and as late as Day 6. ^*^p ≤ 0.05 vs. SA/Veh/P or PQ/Veh/T. Error bars are +/− SEM. *n* = 6–8 rats per study group per time point.

Clinical chemistry and hematology results (Figure 3; Supplementary Tables 1-6) for all rats administered PQ (PQ/Veh/P, PQ/Veh/T, PQ/Bar/P, and PQ/Bar/T) at Day 3 were consistent with prominently decreased food consumption/nutrient intake in alignment with early body weight changes (Figure 1B). These findings included Day 3 reductions in serum triglycerides, alkaline phosphatase (ALP), inorganic phosphate, and blood lymphocyte counts, as well as increased red cell mass relative to SA/Veh/P rats (Figures 3A,B; Supplementary Tables 1-6). Progressive resolution of these findings occurred in both PQ/Veh groups with complete to near-complete resolution by Day 14. Whereas in both PQ/Bar groups, persistent reductions in serum triglycerides and phosphorus, concomitant with reductions in serum glucose and urea, as well as elevations in total cholesterol, occurred with similar magnitudes of change throughout the study (Figures 3A,C,D; Supplementary Tables 1-6). These distinct changes in PQ/Bar groups on Day 6 and Day 14 support persistent calorie deficit (of probably mixed causes) and are consistent with their lower body weights and nighttime body motion relative to both PQ/Veh and SA/Veh/P groups after Day 3 (Figures 1B, 2B, respectively).

Other findings observed on Day 6 were indicative of inflammation. These changes included reductions in serum albumin and albumin/globulin ratio, as well as elevations in total globulin and blood neutrophil count (Days 6 and 14) relative to SA/Veh/P rats (Figures 3E,F; Supplementary Tables 1–6). In PQ/Bar groups only, Day 14 serum globulins were of significantly greater magnitude compared with PQ/Veh and SA/Veh/P groups (Figure 3E). Red cell mass (hemoglobin and hematocrit) was lower relative to PQ/Veh and SA/Veh groups and all other time points for all groups.

**Figure 4:**
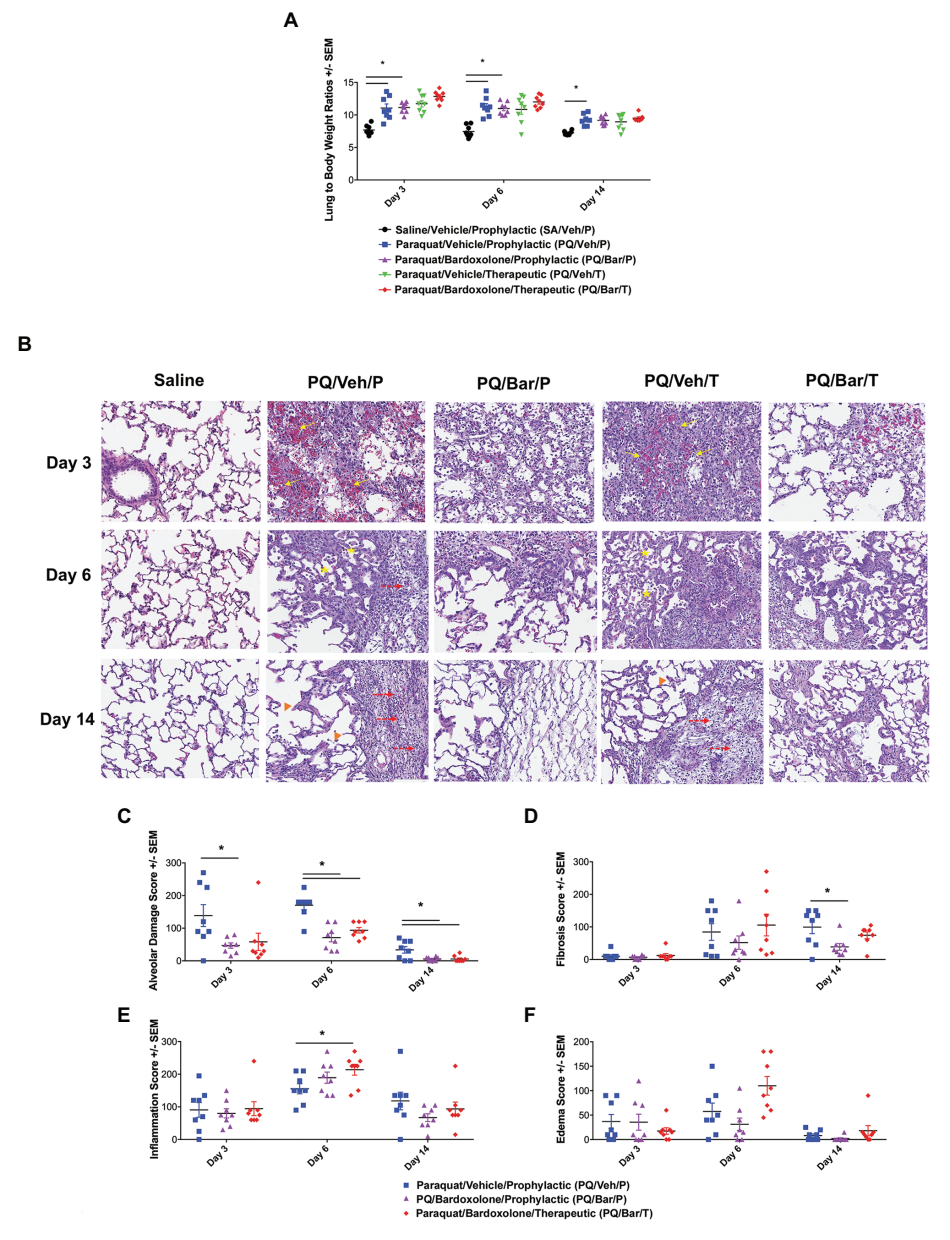
Normalized, endpoint lung weights and histopathology confirmed lung injury and detected effects of bardoxolone treatment. **(A)** Lung weight to body weight ratios. Compared with SA/Veh/P rats, PQ/Veh/P rats showed higher normalized lung weights at all evaluated time points. ^*^p ≤ 0.01 vs. SA/Veh/P. PQ/Bar/P also demonstrated higher normalized weights compared with SA/Veh/P rats on Days 3 and 6. ^*^p ≤ 0.0001 vs. SA/Veh/P. **(B)** Lung histopathology. Lung histopathology confirmed that PQ/Veh/P and PQ/Veh/T rats exhibited alveolar hemorrhage, atelectasis, emphysema, and fibrosis during the course of study. At Day 3, the predominant histological findings were necrosis, loss of type 1 pneumocytes, hemorrhage, and fibrin accumulation within alveoli. Alveolar lumina were multi-focally collapsed and contained abundant hemorrhage, fibrin, and small amounts of necrotic debris (arrows), as well as edema, increased numbers of alveolar macrophages (immune cell infiltrates indicated by stars), and fewer neutrophils. By Day 14, the alveolar septa were expanded by proliferation of cuboidal type II pneumocytes (hyperplasia), fibrin, and varying amounts of fibrous connective tissue (fibrosis indicated by dashed arrows). Furthermore, the alveolar septa were discontinuous with clubbed ends forming large, confluent spaces (emphysema indicated by arrow heads). Perivascular and peribronchiolar connective tissues were expanded by hemorrhage, fibrin, edema, and scattered neutrophils. Rats treated with bardoxolone prophylactically and therapeutically (PQ/Bar/P and PQ/Bar/T, respectively) showed better resolution of alveolar injury. **(C–F)** Histopathology quantification. **(C)** Alveolar damage scores. Compared with PQ/Veh/P rats, PQ/Bar/P possessed higher alveolar damage scores at all evaluated time points, while PQ/Bar/T possessed higher scores on Days 6 and 14. **(D)** Fibrosis scores. Compared with PQ/Veh/P rats, PQ/Bar/P, but not PQ/Bar/T, rats possessed lower fibrosis scores on Day 14. **(E)** Inflammation scores. Compared with PQ/Veh/P rats, PQ/Bar/T, but not PQ/Bar/P, rats possessed higher inflammation scores on Day 6. **(F)** Edema scores. All study groups possessed similar edema scores at all evaluated time points. ^*^p ≤ 0.05 vs. PQ/Veh/P. Error bars are +/− SEM. *n* = 6–8 rats per group per time point.

Serum electrolyte changes generally coincided with changes in breathing rate and pulmonary histopathology. These changes observed on Day 3 included slight elevations in serum bicarbonate (Total CO_2_), without notable change in chloride (except for the PQ/Bar/T rats), sodium, or calculated anion gap (Figures 3G,H; Supplementary Tables 1-6). The pattern was consistent with respiratory acidosis in line with elevations in breathing rate and Day 3 histologic edema (Figures 2A, 4F, respectively). The Day 3 concomitant decrease in chloride in the PQ/Bar/T group was associated with a slight decrease in potassium and suggestive of mixed acid-base change. These changes may have included compensatory response and contraction alkalosis (notably, emesis is not a factor of low serum chloride in rats). Conversely, at Day 6, reductions in bicarbonate and minor elevations in serum chloride occurred in PQ groups (except PQ/Veh/P rats) without notable difference in sodium or calculated anion gap. These Day 6 changes were suggestive of respiratory alkalosis with partial compensation and aligned with the persistent increase in breathing rate observed on Day 6 (Figure 2A). In the PQ/Veh/P rats, the lack of this pattern of change was associated with higher alveolar damage score and relative lung weight (collectively supporting reduced lung volume) compared with the other PQ-administered groups. Other findings in both PQ/Bar groups indicated prominent hepatobiliary injury with increased alanine aminotransferase (ALT), aspartate aminotransferase (AST), and total bilirubin on Day 6 and Day 14 (Figures 3I,J; Supplementary Tables 1-6).

### Normalized Lung Tissue Weights and Histopathology Analyses Also Showed Changes Post-PQ Administration and Response to Bardoxolone Treatment

When lung weights were normalized to body weights, PQ/Veh/P rats showed higher ratios than SA/Veh/P at all evaluated time points (*p* ≤ 0.01; Figure 4A). Similarly, PQ/Bar/P possessed higher normalized lung weights on Days 3 and 6 (*p* ≤ 0.0001 vs. SA/Veh/P). Compared to PQ/Veh rats, PQ/Bar rats showed similar lung to body weight ratios.

Lung histopathology confirmed PQ-induced progressive alveolar injury, hemorrhage, atelectasis (i.e., partial collapse of a lung), emphysema, and fibrosis (Figure 4B). Initially, on Day 3, PQ administration resulted in type 1 pneumocytes loss/necrosis along with hemorrhage and fibrin deposition within alveoli. On Day 6 and Day 14, dead/necrotic type I pneumocytes were replaced by proliferating type II pneumocytes along with thickening of alveolar wall and fibrosis. Atelectasis of airways was also a prominent feature on Day 14. In contrast, PQ/Bar/P and PQ/Bar/T showed reductions in alveolar damage scores at all evaluated time points (*p* ≤ 0.05 for both comparisons vs. PQ/Veh/P; Figure 4C). PQ/Bar/P rats demonstrated reductions in fibrosis scores on Day 14 (*p* ≤ 0.05 vs. PQ/Veh/P), while PQ/Bar/T rats demonstrated reductions in fibrosis scores, although this did not reach statistical significance (Figure 4D). In contrast to alveolar damage and fibrosis, inflammation scores were higher for PQ/Bar/T rats on Day 6 (*p* ≤ 0.05 vs. PQ/Veh/P; Figure 4E). There were no significant differences between treatment groups in severity of pulmonary edema scores (Figure 4F).

### Endpoint Breathing Rate Correlated With Time-Aligned Tissue and Histopathology Measurements

**Figure 5:**
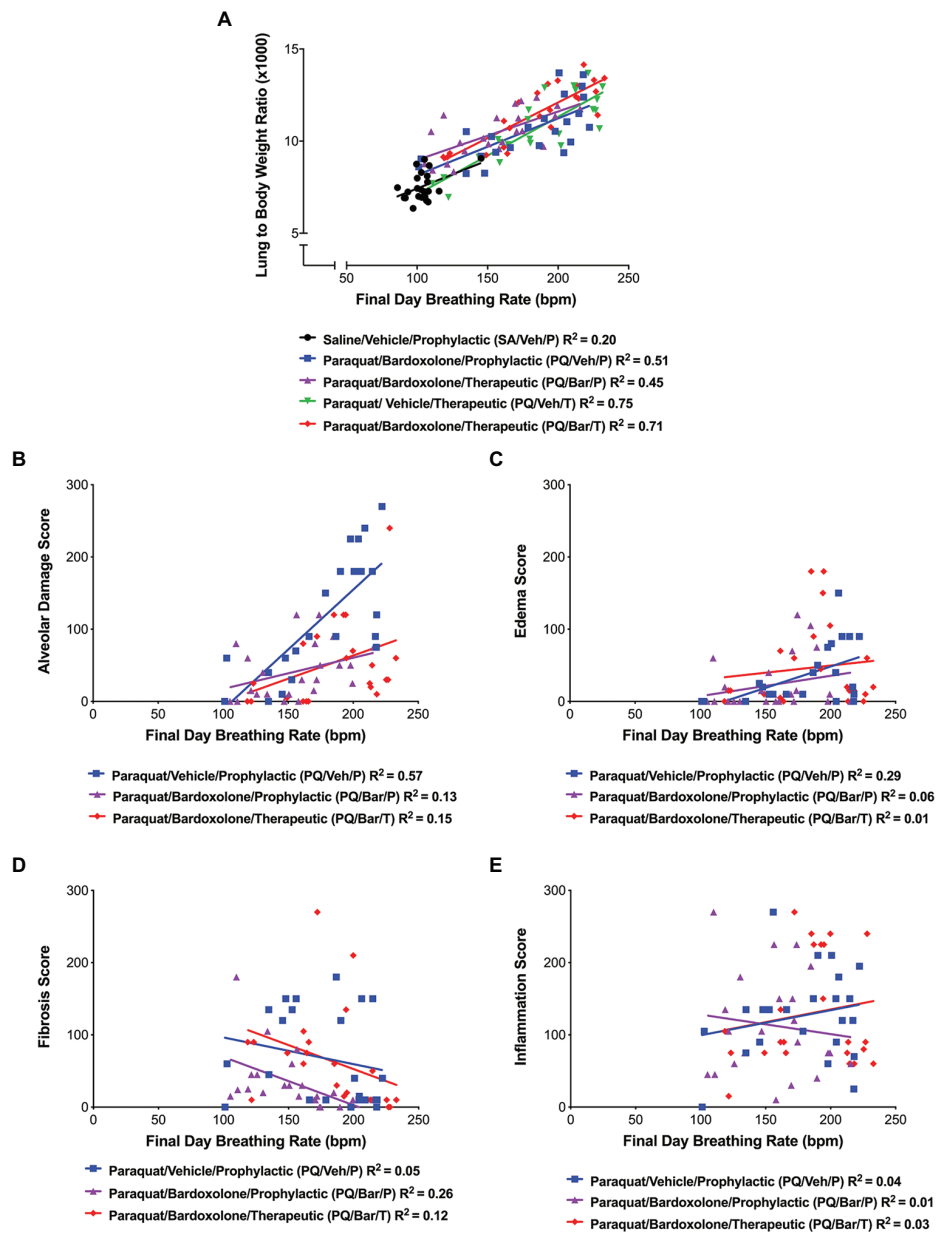
Breathing rate correlated with normalized, endpoint lung weights and histopathology. **(A)** Lung weight to body weight ratios vs. breathing rate. Breathing rate positively correlated with normalized lung weights for all groups as follows: SA/Veh/P (*R* = 0.45), PQ/Veh/P (*R* = 0.71), PQ/Bar/P (*R* = 67), PQ/Veh/T (*R* = 0.87), and PQ/Bar/T (*R* = 0.85); *p* ≤ 0.05. **(B)** Alveolar damage score vs. breathing rate. Breathing rate positively correlated with alveolar damage scores, specifically for rats administered PQ and treated with vehicle (PQ/Veh/P; *R* = 0.76, *p* ≤ 0.0001). **(C)** Edema score vs. breathing rate. Breathing rate positively correlated with edema scores, specifically for PQ/Veh/P rats (*R* = 0.54, *p* ≤ 0.01). **(D)** Fibrosis score vs. breathing rate. Breathing rate negatively correlated with fibrosis scores, specifically for rats administered PQ and treated prophylactically with bardoxolone (PQ/Bar/P; *R* = −0.51, *p* ≤ 0.05). **(E)** Inflammation score vs. breathing rate. There was no significant correlation between inflammation scores and breathing rate for all rats administered PQ in which histopathology quantification was performed. *n* = 22–24 rats per treatment group.

We investigated whether changes in breathing rate correlated with underlying pathology. Endpoint breathing rate positively correlated with normalized lung weights for all groups (*R* = 0.45, *R* = 0.71, *R* = 67, *R* = 0.87, *R* = 0.85 for SA/Veh/P, PQ/Veh/P, PQ/Bar/P, PQ/Veh/T, and PQ/Bar/T, respectively, *p* ≤ 0.05 for all correlations; Figure 5A). In PQ/Veh/P rats only, breathing rate positively correlated with alveolar damage (*R* = 0.76, *p* ≤ 0.0001) and edema scores (*R* = 0.54, *p* ≤ 0.01; Figures 5B,C). The following severity grades were assigned to all histological findings (edema, fibrosis, alveolar damage, and inflammation) during a subjective microscopic evaluation of the H&E-stained liver sections: minimal (1) = less than 20% of area affected, mild (2) = 20% to less than 40% of area affected, moderate (3) = 40% to less than 60% of area affected, marked (4) = 60% to less than 80% of area affected, and severe (5) = more than 80% of area affected. Fibrosis was evaluated by examination of H&E stained lung sections by Pathologist. Special stains like trichrome was not performed since lung has less tissue parenchyma and fibrosis was visible in H&E stained sections. There was a slight negative correlation between breathing rate and fibrosis scores for PQ/Veh/P rats only (*R* = −0.51, *p* ≤ 0.05; Figure 5D). There was no significant correlation between breathing rate and histologic inflammation scores (Figure 5E). Linear regression analyses corroborated these results (Figures 5A–E).

## Discussion

In this study, we investigated the utility of a continuous home cage monitoring platform and its automated breathing rate (BR) and body motion (BM) readouts to detect onset and track injury progression in a rodent model of PQiLI. We also sought to understand the relationship of these digital readouts with standard endpoint measurements (e.g., body weight, body temperature, clinical pathology, and histopathology). Furthermore, we evaluated the sensitivity of BR and BM to detect treatment effects by administering the potentially ameliorative compound bardoxolone either prior to PQ (prophylactic) or following PQ (therapeutic) administration. The results of standard endpoints measurements in this study were consistent with those previously reported for rodent models of PQiLI (Tomita et al., 2007; Xiangdong et al., 2011; Costa et al., 2014; Shao et al., 2015; Rumsey et al., 2017). These included time course effects on body weight, body temperature, lung weights, and lung histologic findings. Similarly, automated BR and BM tracked injury progression over time and enabled a high-resolution method to hourly monitor the initial injury response and severity following PQ administration. Furthermore, the continuous monitoring of these digital readouts enabled a more granular and complete assessment of toxic effects throughout the post-PQ administration study period. Continuous monitoring through both nocturnal and diurnal periods also permitted tracking these physiologic values through a full circadian cycle, optimizing for a clinical translational readout of the animal’s in-life condition. Integrating these digital readouts with standard endpoint measurements, complemented and enhanced interpretation of the latter, such as playing a key role in interpreting the clinical pathology data relevant to respiratory acid-base status and histopathology relevant to differences between intervals.

Due to considerable technical challenges, pulmonary function endpoints in animal studies are not a standardized or routine assessment even with studies of expected pulmonary toxicity or safety testing with inhaled therapeutics (Jenkins et al., 2017). When pursued, these traditional lung function tests, including plethysmography, forced oscillation techniques, and forced ventilator maneuvers, are conducted at limited, pre-determined time points and make a trade-off between snapshots of functional metrics and assessment of the animal in a natural home cage setting with relevant circadian staging. At one end of the spectrum of these traditional tests, the forced oscillation technique (e.g., the flexiVent) captures precise measures of lung pressure, flow, and volume (Hoymann, 2012). This method is invasive, requiring animals to be anesthetized and intubated, as well as laborious, requiring constant monitoring and trained technicians (Vanoirbeek et al., 2010; Hoymann, 2012; Lim et al., 2014). At the other end of the spectrum, less invasive methods of plethysmography, including unrestrained plethysmography, require habituation of animals to the recording chamber in order to gather consistent respiration recordings, as well as to eliminate any stress-related effects of being in the apparatus on breathing rate (Hoymann, 2012; Lim et al., 2014, 2019). Since it is crucial to obtain recordings from a calm rodent, habituation to the plethysmograph chamber has been reported to range from minutes to a week before experimentation, while experiment recordings can range from a few seconds to a couple of hours per animal (Hoymann, 2012; Lim et al., 2014; Quindry et al., 2016). Several studies utilizing plethysmography, forced oscillation techniques or forced ventilator maneuvers in rodent models of lung injury have also reported poor association among related endpoints and inadequate differential in results between affected and control animals (Oury et al., 2001; Vanoirbeek et al., 2010; Milton et al., 2012; Devos et al., 2017; Gilhodes et al., 2017; Jenkins et al., 2017). Hence, a system that allows the evaluation of pulmonary response in the home cage over a full circadian cycle and over a broad study interval has clear advantages not only in the ease of application, but also in accurately reporting the animal’s current condition.

In this study, we demonstrated that continuous, automated monitoring of BR and BM in the home cage can be readily incorporated into a rodent study without impacting standard endpoint measurements. Furthermore, these digital readouts can provide key complementary information regarding pulmonary function, as well the general physiologic state of the animal over the full course of a study. Compared with control rats administered saline, all rats administered PQ showed significant elevations in BR and reductions in nighttime BM the day after induction. These changes persisted until study end for BR and at least until Day 5 (without bardoxolone) or Day 14 (with bardoxolone) for BM, with peak effects around Day 2 for both digital readouts. Elevations in BR were consistent with a previous study of PQiLI in Lewis rats using unrestrained plethysmography, which showed a marked rise in respiratory frequency by Day 3 that persisted to at least Day 7 (Rumsey et al., 2017). Furthermore, BR was shown to be positively correlated with time-aligned lung weights normalized to body weight, as well as alveolar damage and edema histopathology scores, specifically in PQ-instilled rodents not treated with bardoxolone. The additional hepatotoxic effects of bardoxolone may have impacted food consumption and associated body and organ weights, and thus lacked the same correlative findings. Additionally, BR was useful for understanding the converse patterns in serum total CO_2_ and chloride at the acute (Day 3) and subacute (Day 6) intervals by placing it in the context of the relationship between respiration and metabolic acid/base status.

The time course of elevated BR, with peak at Day 2 and gradually decreasing severity for all PQ-administered rats, was consistent with the morphological changes of alveolar damage and edema. In particular, histologic alveolar damage was more severe on Day 3 (acute) and less severe on Day 6 (subacute) and Day 14 (chronic), with study group patterns that generally matched elevations in BR. Furthermore, the PQ study groups with the most/least severe alveolar damage scores and corresponding highest/lowest BR on Day 6 were rats administered PQ and vehicle (PQ/Veh/P) and rats administered PQ and bardoxolone prophylactically (PQ/Bar/P), respectively. Other histologic scores for pulmonary edema and inflammation were generally highest on Day 6 for these groups and were more closely associated with the differential pattern of BR observed among PQ-administered groups on and after Day 6. Elevated lung to body weight ratios, which are consistent with lung edema, were observed in all PQ-administered groups at all evaluated time points and also showed a differential pattern in magnitude among groups that correlated with BR. At the chronic (Day 14) time point, all study endpoints, including BR and BM, showed partial resolution with the exception of the histologic fibrosis score, indicating that these chronic findings and level of fibrosis had less impairment on pulmonary gas exchange than the earlier acute PQ effects in this model. These data are consistent with an initial acute lung injury associated with a severe inflammatory effect in response to PQ treatment peaking around Day 6 after administration and impaired gas exchange due to pulmonary edema. From Day 6, the strong inflammatory and exudative acute lung injury phase started to resolve and is associated with onset of an aberrant fibrotic repair phase characterized by recruitment, expansion, and activation of fibroblasts. Overall, this progression of findings with PQ administration is expected with the model representing acute to subacute edema and inflammation early on, and evolving to alveolar interstitial fibrosis developing as a result of infiltration of myofibroblasts in the alveolar space and septa, differentiation of fibroblasts, and production of collagen (Tomita et al., 2007; Sun and Chen, 2016).

Similar to BR, decreased BM values peaked for all PQ-administered groups at Day 2 and showed gradual resolution thereafter. In addition, differential nighttime BM patterns among PQ groups were closely associated with decreased body weight and clinical pathology changes. In particular, the differential pattern for nighttime BM aligned with liver enzyme and total bilirubin measurements in bardoxolone-treated animals supported bardoxolone-related liver toxicity (as has been previously reported with this product or analog in rats and humans; Pergola et al., 2011; Zoja et al., 2013). Hence, these collective changes were associated with confounding bardoxolone-associated hepatotoxicity. Overall, nighttime BM was a more sensitive and specific measure of overall animal health or injury status better associated with other study findings than daytime body motion, attesting to the value of full-circadian cycle evaluation achieved with the Vium Digital Smart House. In this study, BM findings did not show utility as a functional readout similar to human clinical exercise tests, which are reflective of reduced pulmonary functional capacity (Swigris et al., 2010; Ley et al., 2011; Du Bois et al., 2014).

In the current study, bardoxolone did not ameliorate PQ-induced pulmonary toxicity other than show a potential reduction in histologic alveolar damage scores on Days 3, 6, and 14. This distinction was concomitant with serum chemistry evidence of bardoxolone-related hepatoxicity. Neither the vehicle nor bardoxolone group was used in assessment of the technology; however, since this is a preliminary evaluation of the technology and not of a therapeutic agent. Other standard endpoint measurements, including body weight, body temperature, clinical pathology, and nighttime, BM also showed more severe magnitudes of change with bardoxolone treatment than rats administered PQ alone. These findings with nighttime BM were observed even prior to Day 3 and support the utility of this digital readout for detecting early onset and general toxicity, which is important for assessing therapeutics, not limited to those for pulmonary injury. Bardoxolone, an Nrf2 activator and NF-κΒ suppressor, has been evaluated clinically as a treatment for IPF as well as chronic kidney disease and pulmonary arterial hypertension (Kulkarni et al., 2013; Tayek and Kalantar-Zadeh, 2013; Wang et al., 2015). However, bardoloxone at clinical doses has been shown to have adverse effects in humans, including body weight loss, increased proteinuria, muscle spasms, and liver injury (Tayek and Kalantar-Zadeh, 2013). Bardoxolone-associated reductions in body weight as well as hepatobiliary injury were observed in the current study. These changes included significant elevations in liver-associated serum enzyme activity and total bilirubin observed on Day 6 with worsening of serum bilirubin by Day 14. These results are consistent with previously reported findings in rats administered bardoxolone (Zoja et al., 2013).

There are some limitations to the use of these digital readouts. For example, unlike BR, standard in-life methods to measure pulmonary function provide multiple specific respiratory parameters, such as total breathing cycle time, inspiration/expiration time, tidal volume, and lung mechanics (Hoymann, 2012; Lim et al., 2014). The current system calculates circadian patterns for physical breathing rate alone; however, the value of such is supported in this study, and future improvement of the system may involve extrapolating, as feasible, to a raw readout of chest movements enabling more detailed assessment of breathing cycle.

In conclusion, results of this study demonstrate selective utility of a continuous monitoring platform and its automated BR and BM readouts to detect the complete time course of pulmonary response and general toxicity in a rodent model of PQiLI. This includes endpoints showing the earliest detection of onset, circadian pattern, and the uninterrupted monitoring of pulmonary injury progression. Results of these endpoints complemented standard in-life methods and aligned with underlying morphologic and clinical laboratory pathology. Although BM did not track long-term pulmonary functional impairments in this model, it detected adverse toxic effects of the reference compound bardoxolone. Based on these results, continuous monitoring of digital readouts in the home cage can contribute to more rapid and sensitive detection and comprehensive assessment of pulmonary injury and disease conditions in rodent models.

## Data Availability Statement

The raw data supporting the conclusions of this article will be made available by the authors, without undue reservation.

## Ethics Statement

The animal study was reviewed and approved by Vium’s Institutional Animal Care and Use Committee.

## Author Contributions

SB, AG, AM, DR, SS, DW, and LS were involved in the conception of the study. All authors were involved in the analysis and interpretation of the data. SB, SS, DW, ML, and LS were involved in writing the manuscript. All authors contributed to the article and approved the submitted version.

### Conflict of Interest

The authors declare that this study received funding from Novartis Institutes for BioMedical Research. The funder had the following involvement with the study: the study design, analysis, interpretation of data, the writing of this article, and the decision to submit it for publication. SB, AG, AM, DR, SS, and DW are/were employees and/or stockholders of Novartis, Inc. ML and LS were employees of Vium, Inc. SS is employed by Vertex Pharmaceuticals.

## Footnotes

^1^http://www.growcells.com/
