# Supplementary material for: Continuous, Automated Breathing Rate and Body Motion Monitoring of Rats With Paraquat-Induced Progressive Lung Injury
Source: Front Physiol. 2020 Oct 16;11:569001. doi: 10.3389/fphys.2020.569001 (PMC7596732; doi:10.3389/fphys.2020.569001)
Supplement: Supplementary file 1 [file Table_1.docx]

Supplementary Material

# Supplemental Materials and Methods

Breathing rates measured by computer vision was compared to breathing rate measured by conventional whole-body plethysmography in awake mice with known differences in baseline breathing rates (Murphy, 2002). Six-week old male C57BL/6J (Jackson Labs, Bar Harbor, ME; n = 3) and C3H/HeJ (Jackson Labs, Bar Harbor, ME; n = 3) mice were acclimated to the Smart Housing cages for a total of one week prior to commencing the study. Animals were singly housed three days prior to study start. Unrestrained animals were placed in a whole-body plethysmograph (emka technologies, Falls Church, VA) and the plethysmograph chamber placed inside of the Smart Housing cage to enable the simultaneous collection of data from the plethysmograph and the computer vision algorithms. Video and respiratory traces were collected for 2 hours. For all time ranges in which the computer vision algorithms identified a significant breathing rate, frequency was calculated using the respiratory traces captures on the plethysmograph within an identical 30 sec window. Breathing rate frequencies captured from video and the plethysmograph were highly correlated (Supplementary Figure 2, R2 = 0.981; RMS error = 3.7%). Consistent with the literature, C3H/HeJ mice had a significantly lower breathing rate (136.3 +/- 3.2) than C57BL/6J animals (180.7 +/- 3.7) [ANOVA: F_(1,27)_ = 65.99; *P< 0.0001*]).

# Supplementary Tables

**Supplementary Table 1. Study day 3 clinical pathology measurements for prophylactic treatment groups (Mean +/- STDEV)**

| **Metabolite** | **SA/Veh/P**  **(n=8)** | **PQ/Veh/P**  **(n=8)** | **PQ/Bar/P**  **(n=8)** |
| --- | --- | --- | --- |
| Alanine aminotransferase  (IU/L) | 52.9 ± 7.5 | 39.9 ± 4.6 +C | 56.4 ± 12.6 |
| Aspartate aminotransferase  (IU/L) | 114.6 ± 18.0 | 111.1 ± 21.6 | 127.8 ± 23.7 |
| Alkaline phosphatase  (IU/L) | 203.6 ± 13.5 | 105.0 ± 41.5 #C | 70.1 ± 6.8 #C |
| Total bilirubin  (μmol/L) | 0.85 ± 0.25 | 0.66 ± 0.26 | 1.20 ± 0.28 *D |
| Urea Nitrogen (mmol/L) | 5.88 ± 0.29 | 7.14 ± 0.63 +D | 5.54 ± 0.46 |
| Creatinine (enzymatic method)  (μmol/L) | 19.8 ± 2.6 | 21.3 ± 2.6 | 16.9 ± 2.9 |
| Urea/creatinine ratio  (n/a) | 0.304 ± 0.034 | 0.343 ± 0.064 | 0.336 ± 0.068 |
| Total protein  (g/L) | 57.89 ± 2.74 | 58.54 ± 1.79 | 58.11 ± 1.17 |
| Albumin  (g/L) | 37.45 ± 2.13 | 38.09 ± 1.77 | 38.10 ± 1.33 |
| Globulin  (g/L) | 20.44 ± 1.56 | 20.45 ± 1.45 | 20.01 ± 0.86 |
| Albumin/globulin ratio  (n/a) | 1.84 ± 0.17 | 1.88 ± 0.21 | 1.91 ± 0.15 |
| Glucose (mmol/L) | 7.93 ± 0.59 | 7.58 ± 0.92 | 6.38 ± 0.66 +D |
| Sodium (mmol/L) | 143.1 ± 1.5 | 144.5 ± 1.6 | 144.6 ± 0.7 |
| Chloride (mmol/L) | 97.4 ± 0.9 | 96.4 ± 2.0 | 96.5 ± 0.6 |
| Potassium (mmol/L) | 5.44 ± 0.31 | 5.24 ± 0.29 | 4.94 ± 0.25 |
| Calcium (mmol/L) | 2.55 ± 0.08 | 2.48 ± 0.07 | 2.41 ± 0.08 +D |
| Magnesium (mmol/L) | 0.839 ± 0.038 | 0.890 ± 0.047 *D | 0.914 ± 0.041 +D |
| Total CO_2_ (mmol/L) | 27.99 ± 1.22 | 30.06 ± 2.17 *D | 31.08 ± 1.08 +D |
| Inorganic phosphorus (mmol/L) | 2.39 ± 0.11 | 2.13 ± 0.16 +D | 1.88 ± 0.07 +D |
| Triglycerides (mmol/L) | 1.39 ± 0.18 | 0.73 ± 0.18 +D | 0.93 ± 0.24 +D |
| Total cholestetol (mmol/L) | 2.31 ± 0.22 | 3.40 ± 0.55 +D | 5.36 ± 0.47 +D |
| Creatine kinase (U/L) | 864.6 ± 334.4 | 783.9 ± 344.2 | 990.0 ± 600.2 |
| Total leukocyte count  (x10^9^/L) | 7.73 ± 2.11 | 5.61 ± 1.29*D | 5.79 ± 0.96*D |
| Neutrophil count (%) | 15.45 ± 5.63 | 22.03 ± 3.78 | 20.21 ± 6.48 |
| Lymphocyte count (%) | 82.53 ± 5.79 | 76.04 ± 4.43 | 77.83 ± 7.06 |
| Monocyte count  (%) | 2.03 ± 1.30 | 1.94 ± 1.43 | 2.00 ± 1.31 |
| Erythrocyte count (x10^12^/L) | 9.05 ± 0.43 | 10.02 ± 0.71*C | 9.63 ± 0.25*C |
| Hemoglobin (g/L) | 146.5 ± 6.0 | 163.4 ± 9.4 +D | 155.1 ± 5.5 *D |
| Hematocrit (L/L) | 0.441 ± 0.020 | 0.484 ± 0.031 +D | 0.476 ± 0.017*D |
| Mean cell volume (fL) | 48.63 ± 1.19 | 48.25 ± 1.49 | 49.38 ± 1.19 |
| Mean cell hemoglobin concentration  (g/L) | 333.1 ± 5.4 | 338.8 ± 6.8 | 325.8 ± 6.9 |
| Platelet count (x10^9^/L) | 636.9 ± 240.0 | 911.9 ± 132.0 +D | 853.1 ± 107.5 *D |

+D *P<0.01* vs. SA/Veh/P by Dunnett Test; *D *P<0.05* vs. SA/Veh/P by Dunnett Test; #C *P<0.01* vs. SA/Veh/P by Cochran and Cox Test; +C *P<0.01* vs*.* SA/Veh/P by Cochran and Cox Test; *C *P<0.05* vs. SA/Veh/P by Cochran and Cox Test. SA/Veh/P = Saline/Vehicle/Prophylactic; PQ/Veh/P = Paraquat/Vehicle/Prophylactic; PQ/Bar/P = Paraquat/Bardoxolone/Prophylactic.

**Supplementary Table 2. Study day 6 clinical pathology measurements for prophylactic treatment groups (Mean +/- STDEV)**

| **Metabolite** | **SA/Veh/P**  **(n=8)** | **PQ/Veh/P**  **(n=8)** | **PQ/Bar/P**  **(n=8)** |
| --- | --- | --- | --- |
| Alanine aminotransferase  (IU/L) | 61.1 ± 12.8 | 70.0 ± 18.2 | 419.5 ± 283.2 +C |
| Aspartate aminotransferase  (IU/L) | 105.8 ± 14.8 | 146.5 ± 37.6 *C | 395.8 ± 169.7 +C |
| Alkaline phosphatase  (IU/L) | 203.8 ± 19.7 | 149.6 ± 22.8 +C | 120.3 ± 27.9 +D |
| Total bilirubin  (μmol/L) | 0.94 ± 0.35 | 1.59 ± 0.55 *D | 2.95 ± 0.61 +D |
| Urea Nitrogen (mmol/L) | 6.58 ± 0.26 | 5.86 ± 0.46 | 5.70 ± 1.21 |
| Creatinine (enzymatic method)  (μmol/L) | 20.9 ± 2.1 | 17.4 ± 4.1 | 24.8 ± 5.9 |
| Urea/creatinine ratio  (n/a) | 0.318 ± 0.036 | 0.369 ± 0.161 | 0.233 ± 0.027 +C |
| Total protein  (g/L) | 58.79 ± 1.22 | 56.43 ± 2.63 | 56.60 ± 2.31 |
| Albumin  (g/L) | 39.63 ± 1.20 | 33.50 ± 1.70 +D | 34.11 ± 1.20 +D |
| Globulin  (g/L) | 19.16 ± 0.83 | 22.93 ± 2.58 +C | 22.49 ± 2.01 +C |
| Albumin/globulin ratio  (n/a) | 2.06 ± 0.13 | 1.48 ± 0.21 +D | 1.53 ± 0.16 +D |
| Glucose (mmol/L) | 9.05 ± 1.50 | 7.93 ± 0.83 | 6.64 ± 0.96 +D |
| Sodium (mmol/L) | 143.8 ± 1.2 | 143.5 ± 2.3 | 145.0 ± 0.5 |
| Chloride (mmol/L) | 97.1 ± 0.9 | 97.6 ± 1.6 | 102.0 ± 1.8 +D |
| Potassium (mmol/L) | 5.11 ± 0.21 | 6.08 ± 1.08 *C | 5.17 ± 0.54 |
| Calcium (mmol/L) | 2.73 ± 0.14 | 2.58 ± 0.24 | 2.63 ± 0.29 |
| Magnesium (mmol/L) | 0.826 ± 0.027 | 0.820 ± 0.060 | 1.013 ± 0.054 +D |
| Total CO_2_ (mmol/L) | 25.81 ± 1.50 | 26.14 ± 1.27 | 21.93 ± 1.83 +D |
| Inorganic phosphorus (mmol/L) | 2.18 ± 0.15 | 2.18 ± 0.28 | 1.86 ± 0.25 *D |
| Triglycerides (mmol/L) | 1.89 ± 0.45 | 1.55 ± 0.43 | 0.70 ± 0.15 #C |
| Total cholestetol (mmol/L) | 2.36 ± 0.14 | 2.74 ± 0.30 *C | 5.23 ± 0.59 #C |
| Creatine kinase (U/L) | 578.1 ± 77.6 | 746.5 ± 193.6 | 889.6 ± 388.0 |
| Total leukocyte count  (x10^9^/L) | 9.44 ± 2.46 | 10.87 ± 2.57 | 8.21 ± 1.76 |
| Neutrophil count (%) | 15.29 ± 3.40 | 22.14 ± 3.74 +D | 24.99 ± 3.83 +D |
| Lymphocyte count (%) | 80.15 ± 4.44 | 74.64 ± 5.53 | 71.83 ± 4.78 +D |
| Monocyte count  (%) | 4.58 ± 3.67 | 3.24 ± 2.61 | 3.19 ± 3.55 |
| Erythrocyte count (x10^12^/L) | 9.29 ± 0.24 | 9.89 ± 0.62 *D | 9.21 ± 0.40 |
| Hemoglobin (g/L) | 150.4 ± 4.9 | 159.8 ± 7.4 *D | 149.8 ± 6.1 |
| Hematocrit (L/L) | 0.464 ± 0.016 | 0.491 ± 0.035 | 0.466 ± 0.023 |
| Mean cell volume (fL) | 49.88 ± 1.25 | 49.63 ± 2.00 | 50.75 ± 1.28 |
| Mean cell hemoglobin concentration  (g/L) | 325.4 ± 12.6 | 326.6 ± 13.2 | 321.1 ± 9.3 |
| Platelet count (x10^9^/L) | 664.0 ± 310.2 | 508.3 ± 54.8 | 631.4 ± 56.7 |

+D *P<0.01* vs. SA/Veh/P by Dunnett Test; *D *P<0.05* vs. SA/Veh/P by Dunnett Test; #C *P<0.01* vs. SA/Veh/P by Cochran and Cox Test; +C *P<0.01* vs*.* SA/Veh/P by Cochran and Cox Test; *C *P<0.05* vs. SA/Veh/P by Cochran and Cox Test. SA/Veh/P = Saline/Vehicle/Prophylactic; PQ/Veh/P = Paraquat/Vehicle/Prophylactic; PQ/Bar/P = Paraquat/Bardoxolone/Prophylactic.

**Supplementary Table 3. Study day 14 clinical pathology measurements for prophylactic treatment groups (Mean +/- STDEV)**

| **Metabolite** | **SA/Veh/P**  **(n=6)** | **PQ/Veh/P**  **(n=7)** | **PQ/Bar/P**  **(n=8)** |
| --- | --- | --- | --- |
| Alanine aminotransferase  (IU/L) | 55.5 ± 6.8 | 57.6 ± 4.1 | 209.3 ± 53.0 #C |
| Aspartate aminotransferase  (IU/L) | 114.8 ± 13.8 | 102.1 ± 20.6 | 198.6 ± 54.7 +C |
| Alkaline phosphatase  (IU/L) | 205.2 ± 18.3 | 221.7 ± 13.2 | 189.0 ± 13.6 |
| Total bilirubin  (μmol/L) | 1.08 ± 0.26 | 0.94 ± 0.25 | 4.51 ± 0.96 #C |
| Urea Nitrogen (mmol/L) | 6.60 ± 0.35 | 6.60 ± 0.61 | 5.88 ± 0.77 |
| Creatinine (enzymatic method)  (μmol/L) | 21.5 ± 3.0 | 20.0 ± 1.8 | 18.1 ± 1.5 |
| Urea/creatinine ratio  (n/a) | 0.313 ± 0.050 | 0.331 ± 0.044 | 0.328 ± 0.057 |
| Total protein  (g/L) | 59.25 ± 2.20 | 60.00 ± 1.42 | 67.63 ± 2.30 +D |
| Albumin  (g/L) | 38.60 ± 2.11 | 38.50 ± 1.36 | 40.36 ± 1.87 |
| Globulin  (g/L) | 20.65 ± 0.58 | 21.50 ± 1.08 | 27.26 ± 1.22 +D |
| Albumin/globulin ratio  (n/a) | 1.85 ± 0.12 | 1.79 ± 0.14 | 1.49 ± 0.08 +D |
| Glucose (mmol/L) | 8.98 ± 1.15 | 8.89 ± 1.73 | 6.21 ± 0.89 +D |
| Sodium (mmol/L) | 140.5 ± 1.4 | 142.3 ± 1.9 | 143.0 ± 1.7 *D |
| Chloride (mmol/L) | 96.3 ± 1.0 | 96.8 ± 2.3 | 95.8 ± 2.0 |
| Potassium (mmol/L) | 5.09 ± 0.34 | 4.90 ± 0.22 | 5.23 ± 0.56 |
| Calcium (mmol/L) | 2.50 ± 0.063 | 2.47 ± 0.049 | 2.55 ± 0.053 |
| Magnesium (mmol/L) | 0.843 ± 0.030 | 0.813 ± 0.077 | 0.858 ± 0.06 |
| Total CO_2_ (mmol/L) | 29.43 ± 0.92 | 29.47 ± 1.11 | 28.31 ± 1.42 |
| Inorganic phosphorus (mmol/L) | 2.25 ± 0.10 | 2.33 ± 0.18 | 2.28 ± 0.16 |
| Triglycerides (mmol/L) | 1.52 ± 0.33 | 1.64 ± 0.35 | 1.16 ± 0.33 |
| Total cholestetol (mmol/L) | 2.38 ± 0.15 | 2.59 ± 0.14 | 6.08 ± 0.9 #C |
| Creatine kinase (U/L) | 902.0 ± 340.6 | 663.0 ± 329.7 | 990.0 ±67.79 |
| Total leukocyte count  (x10^9^/L) | 8.41 ± 3.63 | 10.26 ± 1.36 | 10.09 ± 2.41 |
| Neutrophil count (%) | 12.43 ± 4.59 | 17.86 ± 5.37 | 20.88 ± 3.56 +D |
| Lymphocyte count (%) | 81.97 ± 7.28 | 78.01 ± 5.85 | 73.19 ± 4.57 *D |
| Monocyte count  (%) | 5.60 ± 4.41 | 4.16 ± 2.28 | 5.91 ± 5.17 |
| Erythrocyte count (x10^12^/L) | 8.81 ± 2.16 | 9.68 ± 0.47 | 9.36 ± 0.34 |
| Hemoglobin (g/L) | 135.2 ± 34.3 | 149.4 ± 5.4 | 139.8 ± 4.6 |
| Hematocrit (L/L) | 0.422 ± 0.100 | 0.454 ± 0.027 | 0.435 ± 0.014 |
| Mean cell volume (fL) | 47.67 ± 1.03 | 46.86 ± 0.90 | 46.38 ± 0.92 |
| Mean cell hemoglobin concentration  (g/L) | 320.7 ± 7.0 | 329.0 ± 7.5 | 323.0 ± 6.8 |
| Platelet count (x10^9^/L) | 481.7 ± 238.2 | 537.3 ± 171.3 | 685.0 ± 72.2 |

+D *P<0.01* vs. SA/Veh/P by Dunnett Test; *D *P<0.05* vs. SA/Veh/P by Dunnett Test; #C *P<0.01* vs. SA/Veh/P by Cochran and Cox Test; +C *P<0.01* vs*.* SA/Veh/P by Cochran and Cox Test; *C *P<0.05* vs. SA/Veh/P by Cochran and Cox Test. SA/Veh/P = Saline/Vehicle/Prophylactic; PQ/Veh/P = Paraquat/Vehicle/Prophylactic; PQ/Bar/P = Paraquat/Bardoxolone/Prophylactic.

**Supplementary Table 4. Study day 3 clinical pathology measurements for therapeutic treatment groups (Mean +/- STDEV)**

| **Metabolite** | **PQ/Veh/T**  **(n=8)** | **PQ/Bar/T**  **(n=8)** |
| --- | --- | --- |
| Alanine aminotransferase  (IU/L) | 50.1 ± 11.5 | 43.8 ± 15.3 |
| Aspartate aminotransferase  (IU/L) | 193.3 ± 38.9 | 165.4 ± 52.4 |
| Alkaline phosphatase  (IU/L) | 81.5 ± 9.1 | 88.3 ± 11.2 |
| Total bilirubin  (μmol/L) | 0.69 ± 25 | 0.76 ± 0.20 |
| Urea Nitrogen (mmol/L) | 7.19 ± 0.57 | 6.55 ± 0.90 |
| Creatinine (enzymatic method)  (μmol/L) | 19.6 ± 2.0 | 18.4 ± 2.0 |
| Urea/creatinine ratio  (n/a) | 0.370 ± 0.051 | 0.360 ± 0.064 |
| Total protein  (g/L) | 58.40 ± 2.70 | 58.48 ± 1.49 |
| Albumin  (g/L) | 38.69 ± 2.39 | 37.65 ± 1.37 |
| Globulin  (g/L) | 19.71 ± 1.28 | 20.83 ± 2.10 |
| Albumin/globulin ratio  (n/a) | 1.96 ± 0.18 | 1.83 ± 0.24 |
| Glucose (mmol/L) | 8.79 ± 1.15 | 9.06 ± 1.70 |
| Sodium (mmol/L) | 143.3 ± 1.2 | 145.3 ± 1.2 +D |
| Chloride (mmol/L) | 94.7 ± 1.1 | 93.8 ± 1.4 |
| Potassium (mmol/L) | 5.38 ± 0.52 | 4.79 ± 0.48 |
| Calcium (mmol/L) | 2.50 ± 0.13 | 2.66 ± 0.17 |
| Magnesium (mmol/L) | 0.903 ± 0.034 | 0.900 ± 0.029 |
| Total CO_2_ (mmol/L) | 30.80 ± 2.50 | 31.44 ± 0.77 |
| Inorganic phosphorus (mmol/L) | 2.04 ± 0.07 | 1.95 ± 0.13 |
| Triglycerides (mmol/L) | 0.85 ± 0.16 | 1.23 ± 0.20 +D |
| Total cholestetol (mmol/L) | 3.13 ± 0.39 | 4.10 ± 0.60 +D |
| Creatine kinase (U/L) | 966.0 ± 346.8 | 795.8 ± 394.6 |
| Total leukocyte count  (x10^9^/L) | 4.74 ± 1.25 | 3.42 ± 0.42 *C |
| Neutrophil count (%) | 20.13 ± 6.05 | 32.45 ± 6.48 +D |
| Lymphocyte count (%) | 76.43 ± 6.30 | 63.95 ± 7.14 +D |
| Monocyte count  (%) | 3.45 ± 1.95 | 3.58 ± 4.25 |
| Erythrocyte count (x10^12^/L) | 10.09 ± 0.48 | 9.96 ± 0.23 |
| Hemoglobin (g/L) | 165.6 ± 8.0 | 161.4 ± 2.7 |
| Hematocrit (L/L) | 0.479 ± 0.026 | 0.474 ± 0.011 |
| Mean cell volume (fL) | 47.50 ± 1.20 | 47.63 ± 0.74 |
| Mean cell hemoglobin concentration  (g/L) | 346.0 ± 10.5 | 341.1 ± 6. |
| Platelet count (x10^9^/L) | 888.9 ± 75.3 | 862.3 ± 82.2 |

+D *P<0.01* vs*.* PQ/VEH/T by Dunnett Test; *D *P<0.05* vs. PQ/VEH/T by Dunnett Test; #C *P<0.01* vs. PQ/VEH/T by Cochran and Cox Test; +C *P<0.01* vs*.* PQ/VEH/T by Cochran and Cox Test; *C *P<0.05* vs. P PQ/VEH/T by Cochran and Cox Test. PQ/Veh/T = Paraquat/Vehicle/Therapeutic; PQ/Bar/T = Paraquat/Bardoxolone/Therapeutic

**Supplementary Table 5. Study day 6 clinical pathology measurements for therapeutic treatment groups (Mean +/- STDEV)**

| **Metabolite** | **PQ/Veh/T**  **(n=8)** | **PQ/Bar/T**  **(n=8)** |
| --- | --- | --- |
| Alanine aminotransferase  (IU/L) | 64.9 ± 12.5 | 280.9 ± 181.9 *C |
| Aspartate aminotransferase  (IU/L) | 117.4 ± 33.1 | 301.9 ± 123.7 +C |
| Alkaline phosphatase  (IU/L) | 146.9 ± 21.4 | 96.5 ± 13.2 +D |
| Total bilirubin  (μmol/L) | 1.06 ± 0.29 | 1.46 ± 0.34 *D |
| Urea Nitrogen (mmol/L) | 6.26 ± 0.41 | 5.60 ± 1.14 |
| Creatinine (enzymatic method)  (μmol/L) | 20.4 ± 2.4 | 24.1 ± 3.3 *D |
| Urea/creatinine ratio  (n/a) | 0.311 ± 0.030 | 0.238 ± 0.063 +D |
| Total protein  (g/L) | 56.30 ± 2.94 | 56.23 ± 1.71 |
| Albumin  (g/L) | 34.24 ± 3.33 | 34.06 ± 1.79 |
| Globulin  (g/L) | 22.06 ± 1.95 | 22.16 ± 0.89 |
| Albumin/globulin ratio  (n/a) | 1.56 ± 0.27 | 1.54 ± 0.11 |
| Glucose (mmol/L) | 9.36 ± 0.84 | 7.98 ± 1.05 *D |
| Sodium (mmol/L) | 145.4 ± 1.3 | 145.4 ± 1.1 |
| Chloride (mmol/L) | 100.0 ± 1.6 | 102.9 ± 1.4 +D |
| Potassium (mmol/L) | 5.46 ± 0.56 | 5.08 ± 0.70 |
| Calcium (mmol/L) | 2.44 ± 0.19 | 2.61 ± 0.23 |
| Magnesium (mmol/L) | 0.791 ± 0.052 | 0.971 ± 0.061 +D |
| Total CO_2_ (mmol/L) | 25.44 ± 2.87 | 22.26 ± 1.63 *D |
| Inorganic phosphorus (mmol/L) | 1.95 ± 0.14 | 1.74 ± 0.21 *D |
| Triglycerides (mmol/L) | 1.63 ± 0.65 | 1.00 ± 0.31 *D |
| Total cholestetol (mmol/L) | 2.49 ± 0.32 | 5.39 ± 0.47 +D |
| Creatine kinase (U/L) | 594.6 ± 430.4 | 381.5 ± 205.4 |
| Total leukocyte count  (x10^9^/L) | 8.07 ± 2.62 | 7.52 ± 1.78 |
| Neutrophil count (%) | 30.08 ± 22.67 | 22.55 ± 5.34 |
| Lymphocyte count (%) | 66.11 ± 26.06 | 75.39 ± 5.26 |
| Monocyte count  (%) | 3.83 ± 3.59 | 2.09 ± 1.61 |
| Erythrocyte count (x10^12^/L) | 9.62 ± 0.35 | 9.49 ± 0.44 |
| Hemoglobin (g/L) | 159.0 ± 6.7 | 154.9 ± 4.9 |
| Hematocrit (L/L) | 0.454 ± 0.022 | 0.450 ± 0.019 |
| Mean cell volume (fL) | 47.25 ± 1.58 | 47.75 ± 1.67 |
| Mean cell hemoglobin concentration  (g/L) | 350.0 ± 9.8 | 342.9 ± 8.0 |
| Platelet count (x10^9^/L) | 535.0 ± 64.9 | 574.5 ± 86.1 |

+D *P<0.01* vs*.* PQ/VEH/T by Dunnett Test; *D *P<0.05* vs. PQ/VEH/T by Dunnett Test; #C *P<0.01* vs. PQ/VEH/T by Cochran and Cox Test; +C *P<0.01* vs*.* PQ/VEH/T by Cochran and Cox Test; *C *P<0.05* vs. P PQ/VEH/T by Cochran and Cox Test. PQ/Veh/T = Paraquat/Vehicle/Therapeutic; PQ/Bar/T = Paraquat/Bardoxolone/Therapeutic

**Supplementary Table 6. Study day 14 clinical pathology measurements for therapeutic treatment groups (Mean +/- STDEV)**

| **Metabolite** | **PQ/Veh/T**  **(n=8)** | **PQ/Bar/T**  **(n=7)** |
| --- | --- | --- |
| Alanine aminotransferase  (IU/L) | 52.4 ± 4.6 | 136.7 ± 27.8 #C |
| Aspartate aminotransferase  (IU/L) | 94.4 ± 21.2 | 127.3 ± 16.0 +D |
| Alkaline phosphatase  (IU/L) | 181.8 ± 13.1 | 164.9 ± 13.7 *D |
| Total bilirubin  (μmol/L) | 0.78 ± 0.27 | 3.93 ± 1.09 #C |
| Urea Nitrogen (mmol/L) | 5.99 ± 0.60 | 5.31 ± 0.84 |
| Creatinine (enzymatic method)  (μmol/L) | 19.1 ± 1.9 | 19.0 ± 1.6 |
| Urea/creatinine ratio  (n/a) | 0.315 ± 0.039 | 0.284 ± 0.063 |
| Total protein  (g/L) | 59.48 ± 2.38 | 66.79 ± 1.72 +D |
| Albumin  (g/L) | 39.01 ± 1.27 | 41.24 ± 1.76 *D |
| Globulin  (g/L) | 20.46 ± 1.64 | 25.54 ± 1.20 +D |
| Albumin/globulin ratio  (n/a) | 1.93 ± 0.14 | 1.63 ± 0.14 +D |
| Glucose (mmol/L) | 9.69 ± 1.83 | 7.84 ± 1.08 *D |
| Sodium (mmol/L) | 142.4 ± 0.5 | 144.6 ± 1.6 *C |
| Chloride (mmol/L) | 97.6 ± 1.5 | 97.6 ± 1.4 |
| Potassium (mmol/L) | 5.04 ± 0.37 | 5.03 ± 0.39 |
| Calcium (mmol/L) | 2.50 ± 0.00 | 2.63 ± 0.11 |
| Magnesium (mmol/L) | 0.785 ± 0.041 | 0.829 ± 0.049 |
| Total CO_2_ (mmol/L) | 28.94 ± 1.83 | 28.84 ± 2.79 |
| Inorganic phosphorus (mmol/L) | 2.34 ± 0.11 | 2.19 ± 0.14 *D |
| Triglycerides (mmol/L) | 1.81 ± 0.42 | 1.10 ± 0.42 +D |
| Total cholestetol (mmol/L) | 2.68 ± 0.35 | 5.76 ± 0.74 +D |
| Creatine kinase (U/L) | 547.5 ± 290.7 | 415.3 ± 134.6 |
| Total leukocyte count  (x10^9^/L) | 9.21 ± 1.16 | 8.18 ±0.79 |
| Neutrophil count (%) | 16.33 ± 4.96 | 17.70 ± 1.91 |
| Lymphocyte count (%) | 80.04 ± 5.17 | 75.41 ± 5.76 |
| Monocyte count  (%) | 3.63 ± 4.39 | 6.89 ± 5.19 |
| Erythrocyte count (x10^12^/L) | 9.73 ± 0.32 | 9.33 ± 0.25 *D |
| Hemoglobin (g/L) | 149.3 ± 3.8 | 140.1 ± 5.0 +D |
| Hematocrit (L/L) | 0.456 ± 0.014 | 0.433 ± 0.014 +D |
| Mean cell volume (fL) | 46.75 ± 0.71 | 46.29 ± 0.76 |
| Mean cell hemoglobin concentration  (g/L) | 328.1 ± 5.4 | 325.0 ± 6.0 |
| Platelet count (x10^9^/L) | 658.9 ± 71.3 | 699.6 ± 54.3 |

+D *P<0.01* vs*.* PQ/VEH/T by Dunnett Test; *D *P<0.05* vs. PQ/VEH/T by Dunnett Test; #C *P<0.01* vs. PQ/VEH/T by Cochran and Cox Test; +C *P<0.01* vs*.* PQ/VEH/T by Cochran and Cox Test; *C *P<0.05* vs. P PQ/VEH/T by Cochran and Cox Test. PQ/Veh/T = Paraquat/Vehicle/Therapeutic; PQ/Bar/T = Paraquat/Bardoxolone/Therapeutic

# Supplementary Figures


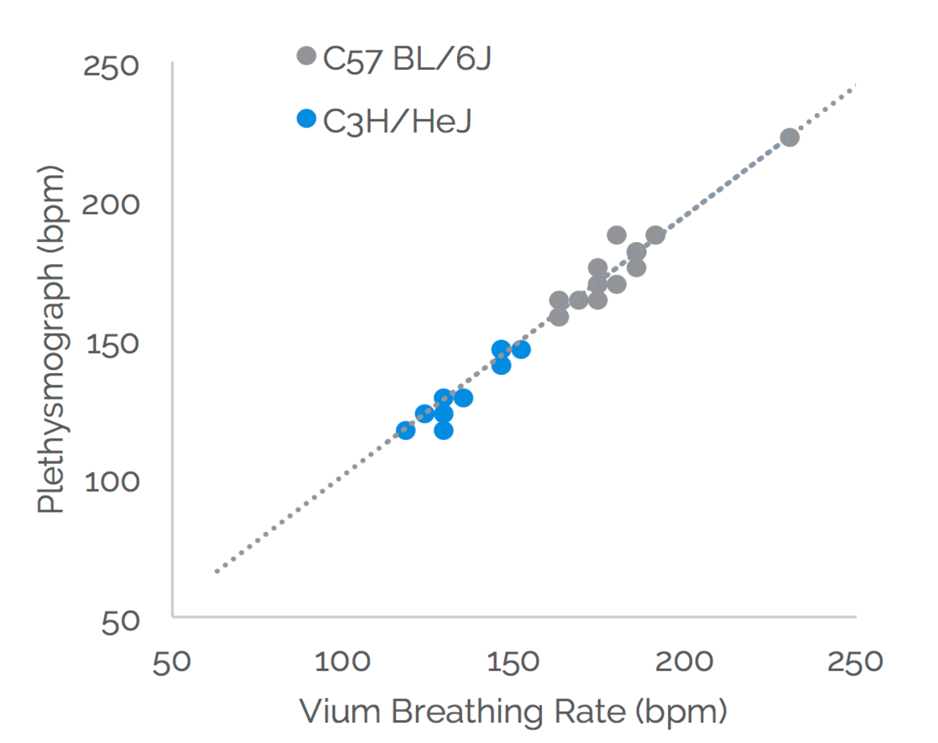


**Supplementary Figure 1. Breathing rates calculated using the computer vision algorithms and standard whole body plethysmography were highly correlated. n=3 animals per group.**

**
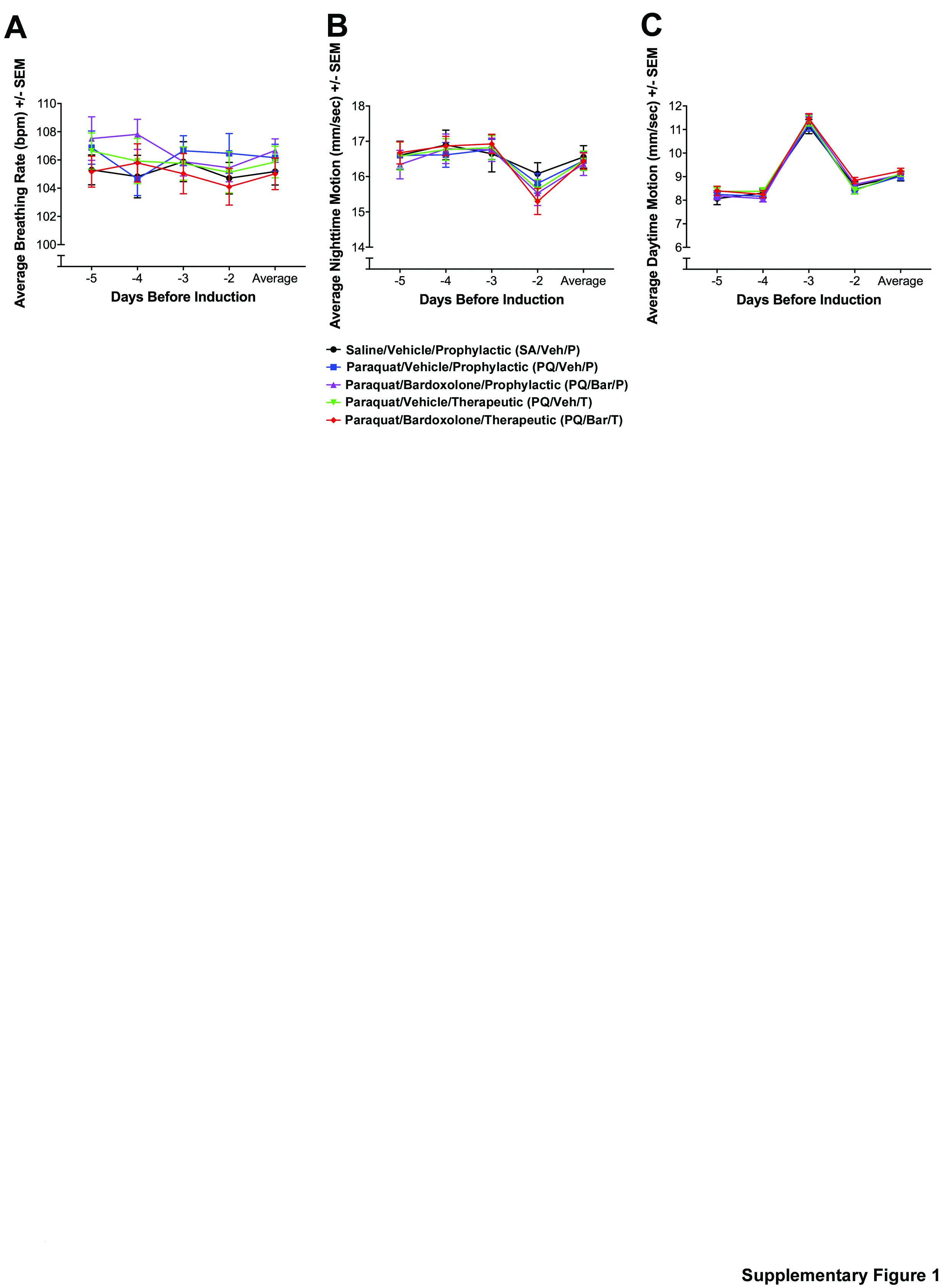
**

**Supplementary Figure 2. Automated breathing rate and body motion provided baseline behavior and physiology.**

Platform provides baseline breathing rate (bpm) **(A),** nighttime body motion (mm/sec) **(B)** and daytime body motion (mm/sec) **(C)** prior to induction. The increase in daytime body motion and decrease in nighttime body motion on Day -2 resulted from a cage change performed on that day. Raw measurements on Days -5 to -2 (days prior to induction) were averaged to acquire a baseline value, which was subtracted from measurements collected after induction. There were no significant differences in baseline measurements among treatment groups, which indicates that subjects were evenly distributed to treatment groups during the randomization procedure.


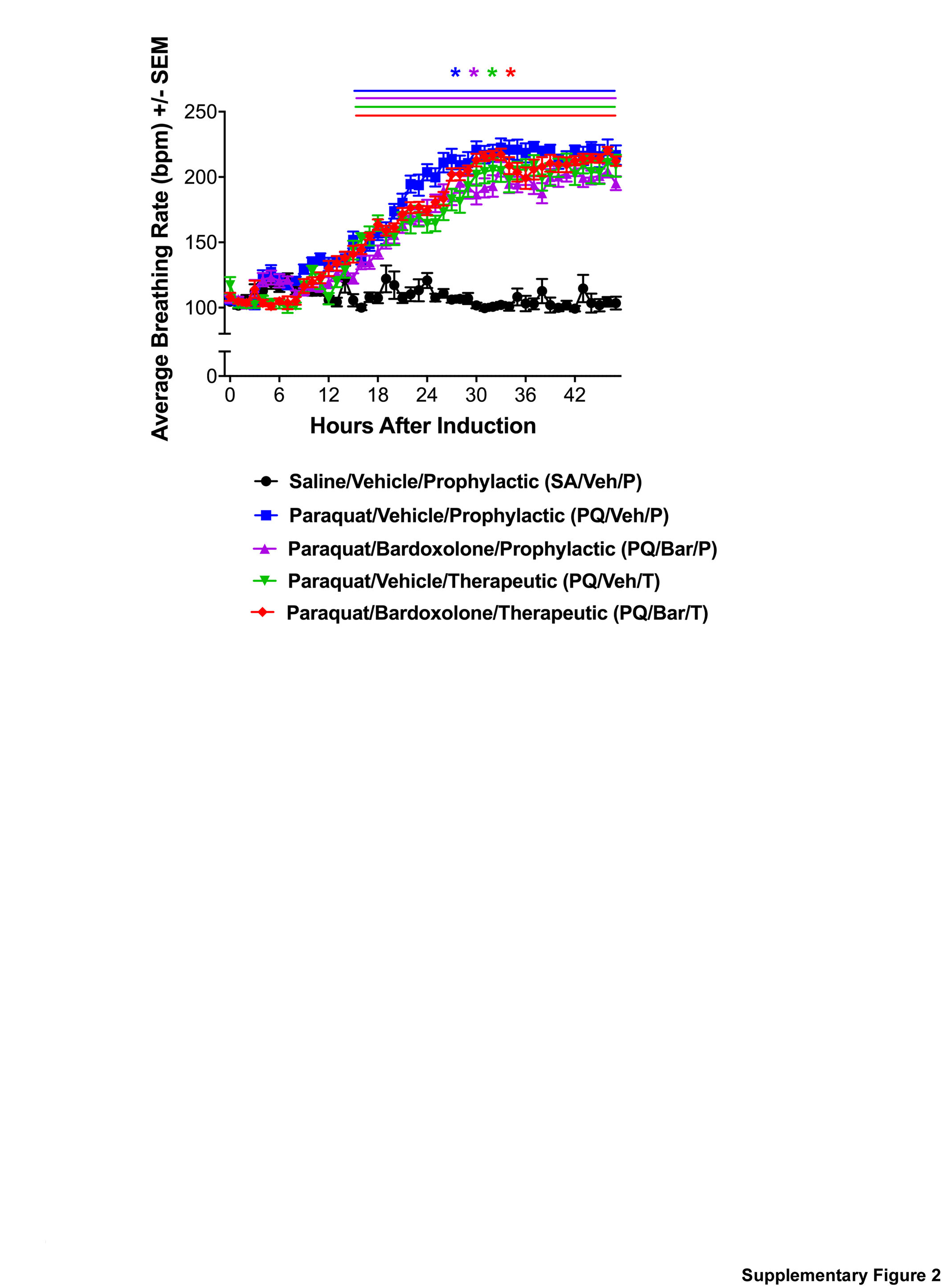


**Supplementary Figure 3. Hourly automated breathing rates captured 24 hours after administration of PQ or saline.**

Compared with SA/Veh/P rats, all rats administered PQ showed consistently elevated breathing rates (bpm) starting 16 hours post-induction (*P<0.0001)*. Bardoxolone-treated rats showed a similar time course compared to vehicle-treated counterparts (n=24 rats for all study groups except n=23 for SA/Veh/P).


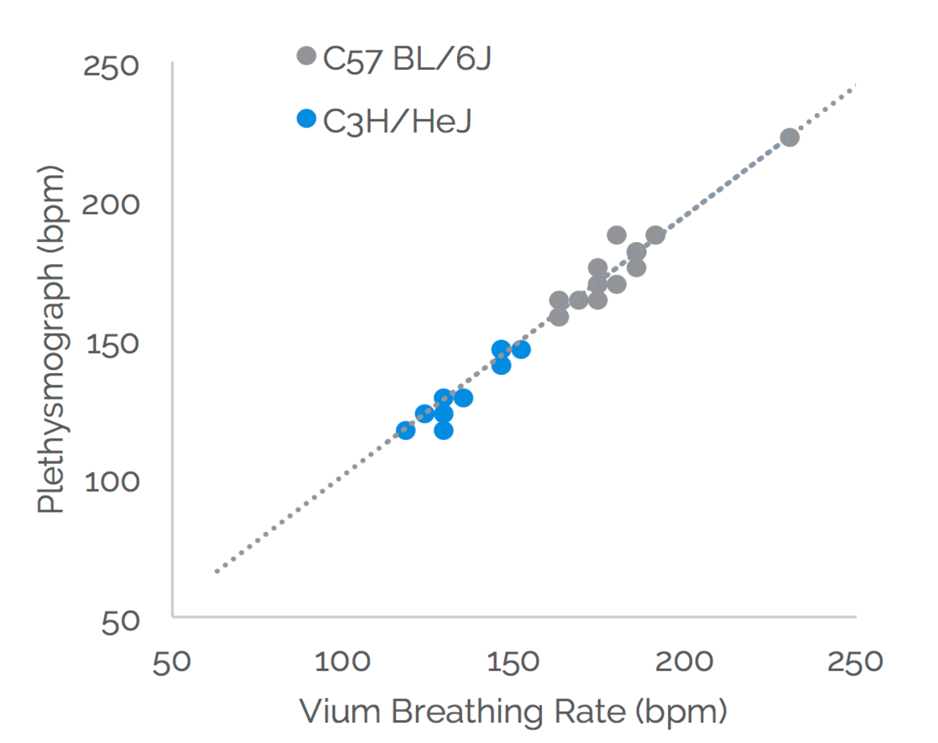


**Supplementary Figure 2. Breathing rates calculated using the computer vision algorithms and standard whole body plethysmography were highly correlated. n=3 animals per group.**

# Supplemental References

Murphy DJ. Assessment of respiratory function in safety pharmacology. Fundam Clin Pharmacol. 2002 Jun;16(3):183-96.
